# The Pharmacology of Bacterial Persistence: From Antibiotic Tolerance to Antimicrobial Resistance

**DOI:** 10.3390/antibiotics15070691

**Published:** 2026-07-16

**Authors:** Maria Cristina Caroleo, Maria Pisano, Erika Cione, Domenica Scumaci, Tommaso Cai, Luca Gallelli, Antonio Leo

**Affiliations:** 1 CRUISE Research Center, Science of Health Department, University Magna Graecia, 88100 Catanzaro, Italy; mariacristina.caroleo@unicz.it; 2 Department of Health Sciences, Magna Græcia University of Catanzaro, Viale Europa-Germaneto, 88100 Catanzaro, Italy; maria.pisano.93@gmail.com (M.P.); aleo@unicz.it (A.L.); 3 Department of Pharmacy, Health and Nutritional Sciences, University of Calabria, 87036 Rende, Italy; erika.cione@unical.it; 4 Research Center on Advanced Biochemistry and Molecular Biology, Department of Experimental and Clinical Medicine, Magna Græcia University of Catanzaro, Viale Europa, 88100 Catanzaro, Italy; scumaci@unicz.it; 5 Department of Urology, Santa Chiara Regional Hospital, 38122 Trento, Italy; tommaso.cai@unitn.it

**Keywords:** antimicrobial resistance, persister cells, antibiotic tolerance, PK/PD, therapeutic drug monitoring, biofilm, MDK_99_, MBEC, VBNC, bacteriophage therapy

## Abstract

**Background/Objectives**: Persistent infections are characterized by recurrent treatment failure and relapse despite apparent antibiotic susceptibility by standard testing, a clinical reality not fully explained by conventional resistance paradigms. Bacterial persister cells, genetically susceptible but phenotypically tolerant subpopulations, survive otherwise lethal antibiotic exposure through reversible physiological adaptations. This review proposes a pharmacological framework linking antimicrobial exposure, bacterial tolerance, persistence, relapse, and the emergence of antimicrobial resistance (AMR). **Methods**: A structured narrative review was conducted using PubMed/MEDLINE, ClinicalTrials.gov, Centers for Disease Control and Prevention (CDC), and World Health Organization/Global Antimicrobial Resistance and Use Surveillance System (WHO/GLASS) sources (inception to 19 June 2026, priority 2010–2026), following Scale for the Assessment of Narrative Review Articles (SANRA) framework principles. **Results**: Resistance, tolerance, and persistence are pharmacologically distinct phenotypes. Persistence occurs without minimum inhibitory concentration (MIC) elevation and is shaped by antimicrobial exposure, target-site penetration, biofilm barriers, intracellular localization, and host stress. The review operationalizes persistence prevention exposure (PPE) in relation to minimum duration for killing 99%/99.99% of cells (MDK_99_/MDK_99.99_), persister fraction, minimum biofilm eradication concentration (MBEC), and biphasic time-kill modeling. **Conclusions**: Antimicrobial therapy should evolve from a model centered solely on MIC suppression and killing of actively growing bacteria toward one that also includes the prevention and eradication of persister reservoirs. Persistence prevention may reduce relapse, repeated antibiotic exposure, and the evolutionary path toward stable AMR.

## 1. Introduction

Antimicrobial resistance (AMR) is one of the most pressing threats to global public health. According to the Centers for Disease Control and Prevention (CDC), more than 2.8 million antimicrobial-resistant infections and over 35,000 deaths occur annually in the United States alone, while global estimates indicate that bacterial AMR contributes to millions of deaths worldwide each year [1,2]. The World Health Organization (WHO), through the Global Antimicrobial Resistance and Use Surveillance System (GLASS), has further highlighted AMR as a major public health and development challenge that requires coordinated surveillance strategies and the optimization of antimicrobial use [3]. The conventional paradigm of AMR is based on a straightforward evolutionary framework: antibiotic exposure imposes selective pressure, resistant mutants survive, and resistant populations progressively emerge and expand through mutation, horizontal gene transfer, clonal expansion, and selection within microbial communities [4,5]. Although this model remains fundamental for understanding the development and dissemination of genetic resistance, it does not fully explain the frequent occurrence of chronic, relapsing, and recurrent infections in which bacterial isolates remain susceptible according to standard antimicrobial susceptibility testing [6,7,8].

**Figure 1:**
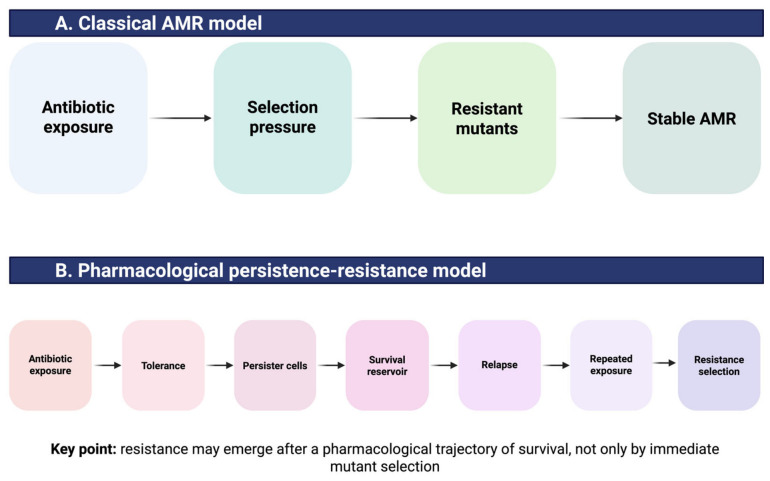
Classical and pharmacological models of antimicrobial resistance. The revised model introduces tolerance and persistence between antibiotic exposure and stable resistance, emphasizing that resistance may emerge after a pharmacological trajectory of survival rather than solely through the immediate selection of pre-existing resistant mutants. Figure created with BioRender (https://www.biorender.com, accessed on 22 June 2026).

The existence of bacterial populations capable of surviving antibiotic treatment despite retaining genetic susceptibility was first recognized by Bigger in 1944, who observed that a small fraction of *Staphylococcus aureus* cells survived exposure to bactericidal concentrations of penicillin [9]. These surviving cells, later termed persisters, have since emerged as a central element in the pathophysiology of difficult-to-eradicate infections and therapeutic failure [7,8]. Persister cells are fundamentally distinct from resistant mutants. They do not carry heritable resistance determinants and typically display antibiotic susceptibility profiles comparable to those of the parental population once active growth resumes. Instead, persisters survive antimicrobial exposure through reversible phenotypic adaptations that transiently reduce their susceptibility to antibiotic killing [6,8]. Current consensus definitions clearly distinguish persistence from resistance, tolerance, and heteroresistance, providing a standardized framework for investigating these related but mechanistically distinct phenomena [6]. Traditionally studied from a microbiological perspective, persistence is increasingly recognized as a phenomenon with profound pharmacological implications. In fact, the survival of persisters often indicates a discrepancy between antibiotic exposure and the physiological state of bacteria: concentrations of drugs and pharmacokinetic–pharmacodynamic (PK/PD) conditions that are highly effective against actively replicating bacteria may prove inadequate for the eradication of dormant, metabolically reprogrammed, biofilm-associated, or otherwise shielded bacterial subpopulations [10,11,12]. From this perspective, persistence can be viewed not merely as a bacterial survival strategy but as a form of pharmacodynamic escape, occurring when antimicrobial exposure fails to engage the biological processes required for bacterial killing [11,13] (Figure 1). This pharmacological interpretation has important clinical consequences. Linking persister biology to antibiotic exposure, target engagement, metabolic activity, and bacterial physiological heterogeneity provides a framework for understanding why apparently appropriate antimicrobial regimens may fail despite the absence of conventional resistance [7,14]. Moreover, it opens new therapeutic opportunities to overcome persistence through exposure optimization, metabolic activation strategies, anti-biofilm approaches, and novel persister-targeting interventions [11,14,15]. In this review, we examine bacterial persistence through a pharmacological lens, integrating current knowledge of persister cell biology with antimicrobial pharmacokinetics and pharmacodynamics, as well as emerging therapeutic strategies. Moreover, we propose a novel conceptual pharmacological framework for interpreting bacterial persistence. Whereas previous reviews have primarily examined persister biology, dormancy, stress responses, and antibiotic tolerance from a microbiological perspective, this analysis proposes a reconceptualization of persistence as a pharmacodynamic and exposure-dependent phenomenon. It integrates persister survival with antimicrobial PK/PD, target-site drug penetration, biofilm and intracellular sanctuaries, therapeutic drug monitoring, relapse prevention, and the evolutionary transition toward stable antimicrobial resistance. Rather than proposing a new validated parameter, this approach develops a conceptual framework that connects persister biology to dosing strategies, treatment failure, and future precision anti-persister therapies.

Additionally, this review distinguishes established pharmacological and microbiological metrics, such as minimum inhibitory concentration (MIC), PK/PD indices, therapeutic drug monitoring (TDM), minimum duration for killing (MDK), minimum biofilm eradication concentration (MBEC), and the mutant selection window, from the conceptual frameworks introduced in this work, including persistence prevention exposure, the persistence window, and the Six-Pillar Pharmacological Persistence Prevention Model. These conceptual frameworks are presented as hypothesis-generating constructs and should not be interpreted as validated clinical parameters or dosing targets. We propose that persistence should be considered not only a microbiological phenomenon but also a critical determinant of antibiotic treatment failure and a potential precursor to the emergence of resistance, thereby representing an essential target for next-generation antimicrobial therapy [6,7,14].

## 2. Methods

This manuscript is a structured narrative review and conceptual pharmacological synthesis. It is submitted as a review rather than as a separate non-standard article category. The phrase “conceptual pharmacological review” is used descriptively to clarify the manuscript focus, not as a journal article type.

### 2.1. Data Sources and Search Window

Searches were performed in PubMed/MEDLINE and complemented by targeted checks in ClinicalTrials.gov, CDC, and WHO/GLASS resources to identify peer-reviewed evidence, registered clinical trials, and institutional surveillance documents relevant to antimicrobial resistance, antibiotic tolerance, bacterial persistence, viable but non-culturable (VBNC) states, and phage-based therapeutic strategies. The search covered database inception to 19 June 2026. Priority was given to studies from 2010 to 2026, but foundational older papers were retained when historically or conceptually essential [8,9]. ClinicalTrials.gov was consulted to identify ongoing or registered interventional studies, while WHO/GLASS and CDC resources were used to contextualize the public health burden of antimicrobial resistance [3,16].

### 2.2. Search Strategy

Representative PubMed/MEDLINE search string: (“persister cells” OR “bacterial persistence” OR “antibiotic persistence” OR “antibiotic tolerance”) AND (“antimicrobial resistance” OR “antibiotic resistance” OR “treatment failure” OR relapse) AND (“pharmacokinetics” OR “pharmacodynamics” OR “PK/PD” OR “therapeutic drug monitoring” OR biofilm OR “combination therapy”).

Additional focused searches were performed for MDK 99%/99.99% of cells (MDK_99_/MDK_99.99_), persister fraction, MBEC, biphasic time-kill modeling, metabolic activation, caseinolytic protease P (ClpP) activators, host-directed therapy, bacteriophage therapy, and artificial intelligence (AI) in antibiotic discovery or resistance prediction [6,17,18,19,20,21,22].

### 2.3. Evidence Identification and Inclusion

Because this was not a systematic review, a full PRISMA-based study-selection process and an exhaustive enumeration of records were not conducted. Although a formal PRISMA flow diagram was not generated, approximately 380 records were initially identified and screened at the title/abstract level across PubMed/MEDLINE and complementary targeted sources. After removing irrelevant, duplicate, non-bacterial, non-pharmacological, or exclusively genetic-resistance-focused records, about 190 articles were assessed in greater detail. Of these, 135 core sources were retained for their direct relevance to bacterial persistence, tolerance, PK/PD, therapeutic drug monitoring, biofilm pharmacology, anti-persister strategies, clinical translation, and antimicrobial resistance.

Study selection was based on the robustness of the definitions, the experimental models used, the quantitative endpoints reported, and the relevance to bacterial persistence or tolerance and to antimicrobial pharmacology. Greater emphasis was placed on studies using standardized terminology, time-kill or regrowth-based endpoints, biofilm or intracellular infection models, dynamic PK/PD systems, or clinically relevant infection settings. Studies focusing solely on genetic resistance mechanisms, on ecological antimicrobial resistance (AMR) surveillance without pharmacological relevance, or on non-bacterial persistence were excluded or deprioritized. Our synthesis adhered to SANRA principles by maintaining a clear rationale, transparent source selection, balanced interpretation of diverse evidence types, and a clear distinction between established metrics and novel conceptual frameworks introduced by authors.

To improve transparency, however, a structured evidence map was generated. Search outputs were screened narratively for conceptual and pharmacological relevance. The final bibliography includes 135 core sources: consensus definitions, mechanistic reviews, experimental studies, translational therapeutic papers, public health reports, clinical trial registrations, and narrative review methodology guidance, all assessed according to the SANRA framework [23] (Table 1).

Inclusion criteria were (1) direct relevance to bacterial persistence, tolerance, or persister cells; (2) pharmacological relevance to antimicrobial exposure, PK/PD, target-site concentration, TDM, biofilm, combination therapy, or anti-persister strategies; and (3) relevance to AMR burden, clinical translation, or trial development. Exclusion criteria were (1) purely ecological AMR studies without pharmacological relevance; (2) non-bacterial persistence unless conceptually necessary; (3) papers focused exclusively on genetic resistance mechanisms without connection to tolerance, persistence, relapse, or treatment failure. Foundational studies defining bacterial persistence as a reversible phenotypic state were also retained [24].

**Table 1:** Structured evidence map of sources included in the narrative review.

Source Category	Search Focus	Use in Manuscript	Representative References
Consensus and definitions	Persistence, tolerance, resistance, MDK metrics	Terminology and conceptual precision	[6,13,19,25,26]
Foundational and mechanistic literature	Persister physiology and chronic infection	Historical and mechanistic background	[7,8,9,24,27]
Experimental pharmacology	Persistence-resistance evolution; metabolic activation; ClpP	Anti-persister mechanisms and proof-of-concept	[18,20,28,29,30]
Biofilm and target-site literature	Biofilm tolerance, MBEC, biofilm PK/PD	Target-site pharmacology and sanctuary model	[12,22,31,32,33]
Therapeutic strategy reviews	Anti-persister drugs, biomaterials, host-directed approaches	Therapeutic framework	[34,35,36,37,38]
Public-health and surveillance sources	AMR burden and GLASS surveillance	Epidemiological context	[1,2,3]
Clinical trials, AI, and clinical decision support	Phage trials, AI antibiotic discovery	AI-driven discovery, clinical resistance prediction, and translational directions	[39,40,41,42,43]
PK/PD, TDM, and antimicrobial exposure optimization	Target attainment, therapeutic drug monitoring (TDM), prolonged infusion, site-of-infection exposure, mutant selection window	Pharmacological optimization of antimicrobial regimens and the connection between exposure, killing, tolerance, and resistance emergence	[31,44,45,46,47]
Narrative-review methodology	Narrative review quality, transparent evidence selection, non-systematic review structure	Methodological justification for evidence mapping and narrative synthesis	[23]

AI, artificial intelligence; AMR, antimicrobial resistance; ClpP, caseinolytic protease P; GLASS, Global Antimicrobial Resistance and Use Surveillance System; MBEC, minimum biofilm eradication concentration; MDK, minimum duration for killing; PK/PD, pharmacokinetic/pharmacodynamic; TDM, therapeutic drug monitoring.

## 3. Resistance, Tolerance, Persistence, and Viable but Non-Culturable (VBNC) States

Bacterial survival during antimicrobial therapy can arise from several distinct biological mechanisms, often grouped under the broad concept of “antibiotic failure.” However, resistance, tolerance, persistence, and VBNC states represent fundamentally different phenomena with important microbiological, pharmacological, and clinical implications [6,13,14]. Antibiotic resistance is typically a genetically encoded trait that enables bacterial growth in the presence of antibiotic concentrations that would inhibit or kill susceptible organisms. Resistance is generally associated with an increase in the MIC and may arise through chromosomal mutations, horizontal gene transfer, or the acquisition of mobile genetic elements carrying resistance determinants [4,5]. From a pharmacological perspective, resistance reflects a shift in the exposure-response relationship, requiring higher antibiotic concentrations to achieve the same antibacterial effect and often resulting in failure to attain PK/PD targets under standard dosing regimens [5,13].

In contrast, antibiotic tolerance does not necessarily involve changes in MIC. Tolerant bacteria remain susceptible by conventional susceptibility testing but exhibit reduced killing kinetics when exposed to bactericidal agents. Therefore, substantially longer exposure times may be required to achieve bacterial eradication [6,13]. Tolerance therefore alters the temporal dimension of antibiotic activity rather than the concentration threshold required for growth inhibition. This distinction has important therapeutic implications because antibiotic regimens optimized to achieve adequate peak concentrations or area-under-the-curve targets may still fail if exposure duration is insufficient to eliminate tolerant populations [11,13].

Persistence represents a specialized form of tolerance characterized by the survival of a small phenotypically distinct subpopulation within an otherwise susceptible bacterial community. Persister cells do not harbor heritable resistance mechanisms and typically regain normal susceptibility once active growth resumes [6,24]. Because most bactericidal antibiotics preferentially target active cellular processes such as cell-wall synthesis, DNA replication, transcription, or protein synthesis, cells that enter a dormant or low-metabolic state become intrinsically less vulnerable to antibiotic-mediated damage [8,15]. Following antibiotic withdrawal, persisters can resume growth and repopulate the infection site, thereby contributing to relapse, chronic infection, and treatment failure despite apparently appropriate antimicrobial therapy [6,14].

The distinction between tolerance and persistence is particularly relevant. While tolerant populations exhibit a uniform reduction in killing rate across the entire bacterial population, persistence involves only a minority of cells entering a protected physiological state [6,13]. Consequently, bacterial killing curves generated during antibiotic exposure typically display biphasic kinetics in the presence of persisters, characterized by rapid elimination of the majority population followed by prolonged survival of a small residual fraction. This phenomenon has become a hallmark of persister cell biology and highlights the limitations of MIC-based susceptibility testing, which provides little information regarding bacterial killing dynamics [6,13,24]. An additional layer of complexity is represented by viable but non-culturable (VBNC) cells. VBNC bacteria remain metabolically active and maintain membrane integrity but fail to grow under routine laboratory culture conditions [25,26]. Unlike classical persister cells, which can rapidly resume proliferation after removal of antibiotic stress, VBNC cells may require specific environmental signals or prolonged recovery periods before regaining culturability [26].

Although persistence and VBNC states were historically considered distinct entities, increasing evidence suggests that they may represent different positions along a continuum of bacterial dormancy. Both phenotypes share profound metabolic downregulation, enhanced stress tolerance, and reduced susceptibility to antimicrobial killing, making precise distinction between them challenging in experimental settings [26,48]. The relationship between persisters and VBNC cells remains an area of active investigation. Some models propose that persisters represent a transient and reversible dormant state, whereas VBNC cells occupy a deeper level of physiological quiescence characterized by severely impaired growth potential [26,49]. Recent studies have suggested that environmental stress, prolonged antibiotic exposure, nutrient limitation, oxidative stress, and host immune pressure may drive transitions between these phenotypic states [25,26,48]. Rather than representing discrete categories, persistence and VBNC formation may, therefore, constitute adaptive survival strategies distributed along a dormancy spectrum [25,26,48]. From a therapeutic perspective, these distinctions are highly relevant.

Conventional antimicrobial development has largely focused on overcoming genetic resistance, whereas tolerant, persister, and VBNC populations challenge treatment efficacy through fundamentally different mechanisms [13,14]. Importantly, increasing antibiotic concentrations may not effectively eliminate dormant cells because the underlying problem is often reduced target activity rather than inadequate drug exposure [8,11]. This observation shifts the focus from susceptibility alone toward bacterial physiological state as a critical determinant of therapeutic success. Consequently, emerging strategies increasingly aim to manipulate bacterial metabolism, promote exit from dormancy, disrupt stress-response pathways, or selectively target non-growing cells [11,14,15].

Understanding the biological and pharmacological differences among resistance, tolerance, persistence, and VBNC states is therefore essential for interpreting treatment failure, designing effective antimicrobial regimens, and developing next-generation anti-infective therapies capable of eradicating bacterial populations that evade conventional antibiotic killing despite retaining genetic susceptibility [6,14] (Table 2).

## 4. Pharmacological Determinants of Persistence

The emergence and survival of persister cells are traditionally viewed as consequences of bacterial stress adaptation and phenotypic heterogeneity. However, increasing evidence suggests that persistence should also be understood as a pharmacological phenomenon arising from the dynamic interaction between bacterial physiology, antimicrobial exposure, and the infection microenvironment [15,50]. In this framework, persister survival is not solely determined by intrinsic bacterial properties but by the inability of a given antibiotic regimen to achieve effective target engagement across all bacterial subpopulations. Mechanistic studies have identified multiple biological pathways involved in persister formation, including metabolic downregulation, activation of toxin–antitoxin modules, stringent-response signaling, oxidative-stress adaptation, alterations in ATP homeostasis, and transitions toward dormant physiological states [13,34,51,52,53,54]. These biological pathways define the physiological state of persister-prone cells, whereas PK/PD variables determine whether antibiotic exposure is sufficient to engage those cells at the relevant site and for an adequate duration. Thus, the link between bacterial stress-response mechanisms and pharmacokinetic variability should be understood as a continuum of target engagement rather than as two unrelated processes.

Importantly, these mechanisms do not operate in isolation. Their impact on bacterial survival is strongly influenced by local drug concentrations and by the physicochemical conditions encountered at the site of infection [31,50].

From a pharmacological perspective, persistence emerges when antimicrobial exposure is sufficient to eliminate actively replicating bacteria but insufficient to eradicate bacterial populations characterized by reduced metabolic activity, limited antibiotic accessibility, or altered physiological states [11,13]. In such circumstances, antibiotics may achieve nominal PK/PD targets while failing to exert bactericidal activity against dormant or protected cells [31,44]. This concept challenges the traditional assumption that achievement of plasma PK/PD targets necessarily translates into microbiological eradication. Suboptimal drug concentrations, particularly prolonged exposure within sub-MIC ranges, may promote bacterial survival and adaptation without inducing complete killing [45,55]. Delayed attainment of therapeutic concentrations, inadequate loading doses, intermittent exposure patterns, premature treatment discontinuation, and insufficient time above the MIC may further increase the probability of persister survival [44,46]. These factors are especially relevant for time-dependent antibiotics, whose efficacy relies on sustained target exposure throughout the dosing interval [46]. Once bacterial populations have entered persister-prone physiological states, patient-specific pharmacokinetic abnormalities may further widen the mismatch between administered dose, plasma exposure, target-site concentration, and bacterial killing.

Host-related pharmacokinetic variability also contributes substantially to persistence. Critically ill patients frequently exhibit profound alterations in drug disposition, including augmented renal clearance, expanded extracellular volume, hypoalbuminemia, altered tissue perfusion, and extracorporeal drug removal [44]. These changes may result in antibiotic concentrations that are adequate in plasma but insufficient at the site of infection [31]. Consequently, bacterial populations residing within poorly penetrated tissues may experience prolonged exposure to sublethal antibiotic concentrations, creating favorable conditions for persister selection and survival [31,50].

Tissue pharmacology represents another critical but often overlooked determinant of persistence. The concentration of an antimicrobial agent within infected tissues may differ markedly from that measured in systemic circulation [31]. Abscesses, necrotic lesions, biofilms, foreign-body-associated infections, and intracellular niches create complex spatial gradients of antibiotic exposure [12,31,50].

In many cases, bacteria are exposed to concentrations substantially lower than those predicted by plasma pharmacokinetics. Such microenvironmental heterogeneity creates pharmacological sanctuaries in which persister cells can survive despite apparently adequate systemic therapy. Low pH, hypoxia, nutrient deprivation, oxidative stress, high bacterial density, and inflammatory tissue damage can profoundly alter bacterial physiology and antimicrobial efficacy [15,50]. Many bactericidal antibiotics require active bacterial metabolism to exert their lethal effects [15,22]. Therefore, environmental conditions that suppress growth or reduce metabolic activity may indirectly promote persistence by limiting antibiotic target engagement. This phenomenon is particularly evident in chronic infections, where bacteria frequently occupy nutrient-restricted and oxygen-poor environments that favor dormancy and phenotypic heterogeneity [7,50].

Biofilm-associated infections are a clinically relevant example. Within biofilms, bacteria experience steep gradients of oxygen, nutrients, and antibiotic penetration [12,32]. Cells located in deeper biofilm layers often exhibit reduced growth rates, diminished metabolic activity, and increased stress-response activation, creating ideal conditions for persister formation. Importantly, the reduced susceptibility of biofilm-associated bacteria is attributable not only to impaired antibiotic diffusion but also to profound physiological reprogramming that reduces antibiotic-mediated killing [12,33].

Numerous pathogens, including *Staphylococcus aureus*, *Salmonella enterica*, *Mycobacterium tuberculosis*, and other intracellularly adapted or facultative intracellular pathogens (e.g., *Pseudomonas aeruginosa*), can occupy intracellular compartments characterized by acidic pH, limited nutrient availability, and restricted antibiotic penetration [50]. In these environments, bacterial metabolic activity is often reduced, further compromising the efficacy of conventional antimicrobial therapies and facilitating long-term survival [19,50].

A critical implication of this pharmacological perspective is that persistence may frequently remain undetected in routine clinical practice. Standard antimicrobial susceptibility testing is performed under optimized laboratory conditions and primarily identifies genetic resistance by determining MICs. However, MIC measurements provide limited information regarding bacterial killing kinetics, phenotypic heterogeneity, dormancy depth, or the capacity of bacterial subpopulations to survive prolonged antibiotic exposure [6,13]. As emphasized by Huemer and colleagues, persistence is therefore likely to be unrecognized and underestimated in clinical microbiology, despite its potential contribution to treatment failure and infection relapse [14].

Beyond its immediate clinical consequences, persistence may also facilitate the evolution of genetic resistance. By allowing bacterial populations to survive antibiotic exposure for extended periods, persister cells create a reservoir from which resistant mutants can emerge. Experimental studies by Windels et al. demonstrated that persistence can accelerate the evolution of resistance by increasing the survival window during which adaptive mutations accumulate [28]. Similarly, Eisenreich and colleagues highlighted the mechanistic interplay among persistence, stress-response activation, DNA repair systems, and mutagenesis pathways that may ultimately promote the acquisition of stable resistance [29]. Taken together, these observations support a conceptual shift in which persistence is viewed not merely as a bacterial survival phenotype but as the consequence of a pharmacological mismatch between antimicrobial exposure and bacterial physiological state. In this model, treatment failure arises not only because bacteria are resistant, but because antibiotic exposure fails to effectively engage the biological processes required for bacterial killing. Understanding and correcting this mismatch may be one of the most promising strategies for improving antimicrobial efficacy and preventing both persistence-driven relapse and the emergence of subsequent resistance.

### Pathogen-Specific Determinants of Persistence

Mechanisms underlying persister survival vary significantly among pathogens, resulting in distinct pharmacological consequences [34,56]. In Gram-negative rods such as *Pseudomonas aeruginosa* and *Klebsiella pneumoniae*, persistence is influenced by outer-membrane permeability barriers, efflux systems, nutrient and oxygen gradients, quorum-sensing networks, stringent-response pathways, and biofilm architecture [12,33,57,58,59]. In *P. aeruginosa*, chronic airway and device-associated infections are characterized by profound spatial heterogeneity, mucoid biofilm formation, low-oxygen microdomains, quorum-sensing-dependent persister formation, and metabolically diverse subpopulations, all of which reduce antibiotic target engagement and favor survival during treatment [12,33,57,58]. In *K. pneumoniae*, capsule production, biofilm formation, carbapenemase associated treatment complexity, and dense bacterial communities may further amplify local exposure heterogeneity and phenotypic tolerance, particularly in device-associated, urinary, pulmonary, or intra-abdominal infections [59,60,61,62].

Gram-positive cocci, such as *Staphylococcus aureus,* frequently develop persistence through mechanisms including slow-growth states, small-colony variants, intracellular survival, toxin–antitoxin and stress-response pathways, metabolic remodeling, and biofilm formation on prosthetic material [63,64,65,66,67]. In staphylococcal infections, persistence is therefore frequently associated with intracellular reservoirs, osteoarticular infections, endocarditis, abscesses, and foreign-body-associated biofilms [63,64,65,66,67]. These differences are clinically relevant because the pharmacological solution is unlikely to be identical across pathogens. For Gram-negative biofilm-associated infections, optimization may require attention to penetration barriers, efflux, local oxygen/nutrient gradients, and anti-biofilm or phage-based strategies [12,33,59,60]. In contrast, effective anti-persister strategies for S. aureus may necessitate enhanced intracellular activity, rigorous source control, biofilm-active combinations, and approaches targeting slow-growing or small-colony-variant populations [63,64,65,66]. Therefore, the proposed persistence-oriented PK/PD framework should be applied as a pathogen- and niche-specific model rather than as a universal bacterial paradigm.

## 5. PK/PD Determinants and the Operationalization of Persistence Prevention Exposure (PPE)

The modern pharmacology of antimicrobial therapy is largely based on the integration of pharmacokinetic (PK) and pharmacodynamic (PD) principles [44,68,69,70]. Classical PK/PD indices, including the fraction of time that free drug concentrations remain above the minimum inhibitory concentration (fT > MIC) for β-lactams, the area under the concentration-time curve to MIC ratio (AUC/MIC) for concentration-dependent agents, and the peak concentration to MIC ratio (Cmax/MIC) for aminoglycosides, have substantially improved antibiotic dosing strategies and clinical outcomes [44,68,69]. These indices remain indispensable for optimizing antibacterial activity and minimizing the emergence of resistance [44,45]. Despite their proven clinical value, conventional PK/PD targets share a common limitation: they are fundamentally derived from MIC-based susceptibility testing and primarily reflect the response of actively growing bacterial populations [6,21]. Consequently, they provide limited information regarding the survival of dormant cells, the eradication of persister subpopulations, antibiotic activity within biofilms, intracellular bacterial persistence, or the probability of bacterial regrowth after treatment discontinuation [8,12,14,50].

**Figure 2:**
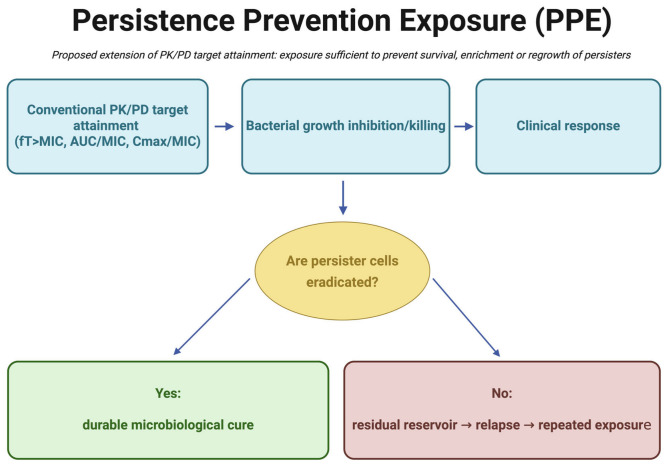
PPE as an extension of conventional PK/PD target attainment. Conventional PK/PD indices (fT > MIC, AUC/MIC, and Cmax/MIC) optimize bacterial killing but do not account for the persister subpopulation. When persisters are eradicated, durable microbiological cure is achieved; when they survive, a residual reservoir drives relapse and repeated exposure. PPE is proposed to close this gap, defining the exposure required to prevent persister survival and regrowth beyond conventional bactericidal endpoints. AUC, area under the concentration-time curve; Cmax, maximum plasma concentration; fT > MIC, free drug time above the minimum inhibitory concentration; MIC, minimum inhibitory concentration; PK/PD, pharmacokinetic/pharmacodynamic; PPE, persistence prevention exposure. Figure created with BioRender (https://www.biorender.com, accessed on 22 June 2026).

Successful attainment of traditional PK/PD targets does not necessarily guarantee complete bacterial eradication. Clinical relapse may occur even when antibiotic exposure exceeds established PK/PD thresholds and susceptibility testing predicts microbiological success [7,14]. Such discrepancies suggest that additional pharmacodynamic dimensions, beyond growth inhibition and immediate bacterial killing, must be considered when evaluating treatment efficacy. To address this challenge, we propose PPE as a complementary pharmacological framework for characterizing antibiotic exposures that prevent the survival, enrichment, and post-treatment recovery of persister populations (Figure 2).

Importantly, PPE is not intended to replace existing PK/PD metrics. Rather, it represents an integrative concept that combines established measures of bacterial susceptibility and killing kinetics with parameters specifically related to persistence.

Recent advances in persister research have provided several quantitative tools that can help operationalize PPE. Among these, the MDK metrics proposed by Brauner and colleagues represent a particularly important development. Unlike MIC, which quantifies growth inhibition, MDK_99_ and MDK_99.99_ measure the time required to eliminate 99% and 99.99% of a bacterial population, respectively. These parameters enable discrimination between resistance and tolerance and provide valuable information regarding bacterial killing dynamics [6,13,21]. Despite their potential, the clinical implementation of MDK_99_ and MDK_99.99_ remains limited. Most clinical microbiology laboratories focus on concentration-based susceptibility thresholds, such as MIC determination and categorical breakpoint interpretation, rather than standardized time-kill assays. Measuring MDK endpoints requires multiple samples, cell viability counting, longer incubation times, and complex analysis, which makes them impractical for routine diagnostics. Consequently, MDK_99_ and MDK_99.99_ should currently be considered research-oriented and preclinical pharmacodynamic tools that inform experimental models of tolerance and persistence, rather than standard clinical microbiology endpoints. Standardization, automation, and validation in dynamic infection models will be necessary before these parameters can be integrated into clinical decision-making.

**Figure 3:**
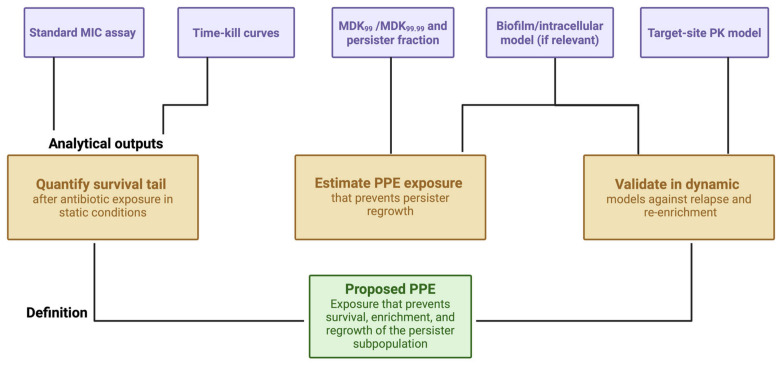
Operationalizing PPE. PPE is derived by integrating five experimental inputs (MIC assay, time-kill curves, MDK99/MDK99.99, persister fraction, and target-site PK modeling) into three analytical steps: quantification of the survival tail, estimation of the regrowth-preventing exposure, and validation in dynamic models. The resulting PPE defines the antibiotic exposure required to prevent persister survival, enrichment, and regrowth, establishing it as a testable preclinical construct distinct from conventional PK/PD indices. MDK, minimum duration of killing; MIC, minimum inhibitory concentration; PK/PD, pharmacokinetic/pharmacodynamic; PPE, persistence prevention exposure. Figure created with BioRender (https://www.biorender.com, accessed on 24 June 2026).

However, MDK values alone do not fully capture the complexity of persistence because they do not directly address bacterial regrowth following antibiotic withdrawal. Similarly, quantification of persister fractions, biphasic killing kinetics, and biofilm-associated survival provides important but incomplete information [6,14]. Biofilm studies frequently employ the MBEC, which better reflects the antibiotic exposure required to eliminate sessile bacterial populations than conventional MIC testing [18,32]. Yet even MBEC measurements may fail to predict post-treatment bacterial recovery if surviving persister cells remain viable [33,54]. Within this framework, PPE may be operationally defined as “the antibiotic exposure, measured within a defined experimental or clinical model, that prevents the survival, enrichment, or post-exposure regrowth of persister subpopulations under specified infection-site conditions.” This definition intentionally incorporates both microbiological and pharmacological dimensions. Herein, PPE is introduced as a conceptual and preclinical idea. It is not yet a validated PK/PD index, does not have a set clinical breakpoint, and should only be used as a dosing target in experimental or model research settings. Unlike conventional susceptibility endpoints, PPE explicitly considers the infection microenvironment, bacterial physiological state, tissue drug penetration, and the potential for relapse after cessation of therapy [31,50]. It must be acknowledged, however, that PPE lacks validated clinical breakpoints and cannot currently be measured by a single standardized assay. In preclinical settings, its estimation requires integration of multiple complementary parameters, as operationalized in Figure 3 and Table 3, and prospective experimental studies will be required before PPE thresholds can be translated into routine dosing guidance.

In preclinical settings, PPE could be estimated through the integration of multiple complementary parameters:

1. MIC determination, providing the baseline susceptibility profile;
2. Time–kill kinetics, including characterization of biphasic killing patterns;
3. MDK_99_ and MDK_99.99_ measurements, quantifying bacterial killing dynamics;
4. Persister fraction analysis following standardized antibiotic exposure;
5. MBEC or other biofilm-eradication metrics when biofilm-associated infection is relevant;
6. Target-site pharmacokinetic simulations, incorporating tissue penetration and local drug exposure [31];
7. Post-treatment regrowth assays, evaluating bacterial recovery after antibiotic withdrawal.

A critical distinction between PPE and traditional PK/PD endpoints is that the primary outcome is not simply bacterial reduction during treatment but the suppression of bacterial resurgence after treatment discontinuation [6,14]. From this perspective, the absence of regrowth serves as a pharmacologically meaningful endpoint, reflecting the effective eradication of both actively growing bacteria and dormant persister populations [7,8]. The relevance of PPE becomes particularly evident in chronic and difficult-to-treat infections. In biofilm-associated infections, prosthetic-device infections, osteomyelitis, cystic fibrosis lung disease, tuberculosis, and intracellular bacterial infections, therapeutic failure frequently reflects the persistence of viable bacterial reservoirs despite apparently adequate antimicrobial exposure [7,12,33,50,71]. In such scenarios, conventional PK/PD target attainment may overestimate therapeutic success because it does not account for persister survival [14,31].

Conceptually, PPE shifts the focus of antimicrobial pharmacology from bacterial killing alone toward durable bacterial eradication. Whereas classical PK/PD indices answer the question, “Was the exposure sufficient to inhibit or kill susceptible bacteria?” PPE addresses a more clinically relevant question: “Was the exposure sufficient to prevent bacterial recovery after therapy?” Although PPE remains a conceptual framework requiring prospective validation, it provides a useful model for integrating microbiological, PK, PD, and infection-microenvironmental determinants of persistence [15,31,50]. Future experimental studies that combine target-site PK measurements, dynamic infection models, and regrowth-based endpoints may enable the development of pathogen- and antibiotic-specific PPE thresholds [6,31,46]. Such an approach could ultimately support a new generation of PK/PD-guided antimicrobial strategies aimed not only at preventing resistance but also at preventing persistence-driven relapse [14,44]. The future validation of PPE and PW will require a systematic translational approach. In preclinical studies, these constructs may be evaluated using standardized time-kill assays, MDK_99_/MDK_99.99_ measurements, quantification of the persister fraction, post-antibiotic regrowth assays, biofilm models, intracellular infection models, and dynamic PK/PD systems, such as hollow-fiber infection models. These methodologies would enable investigators to determine whether specific antibiotic exposures prevent persister survival and regrowth under controlled conditions at the infection site. Subsequent translational studies should incorporate target-site pharmacokinetics, therapeutic drug monitoring, microbiological relapse endpoints, and model-informed precision dosing. At present, PPE and PW are not validated as clinical dosing targets, but they may offer a future framework for optimizing therapy in infections that are difficult to eradicate, including those that are biofilm-associated, prosthetic-device related, osteoarticular, intracellular, and recurrent.

## 6. Persistence Window (PW) and Mutant Selection Window (MSW)

The MSW is one of the most influential concepts in antimicrobial pharmacodynamics. Originally developed to explain the emergence of resistance during antibiotic therapy, the MSW describes the range of antibiotic concentrations between the MIC and the mutant prevention concentration (MPC) within which resistant mutants may be selectively enriched. Antibiotic exposures within this range suppress susceptible bacteria while permitting the expansion of less susceptible variants, thereby promoting the evolution of resistance [45,55]. Unlike the MSW, which is an established pharmacodynamic concept supported by experimental and translational evidence, the PW is introduced only as a theoretical idea to define exposure conditions that favor persistence. The PW does not represent a validated concentration range and cannot currently be measured or used in clinical practice.

Although the MSW framework has substantially advanced the understanding of the emergence of resistance, it focuses primarily on genetically resistant populations and does not explicitly account for phenotypic survival mechanisms such as persistence [6,13]. Growing evidence suggests that bacterial survival during therapy cannot always be explained by resistance alone, highlighting the need for complementary pharmacodynamic concepts that capture persistence-related treatment failure [7,14].

Conceptually, the PW represents the range of exposure conditions under which susceptible bacterial populations are substantially reduced, yet persister cells survive and retain the capacity to resume growth after treatment discontinuation. Whereas the MSW identifies conditions that favor the enrichment of resistant mutants, the PW identifies conditions that favor persistence despite apparent microbiological success. Unlike the MSW, which has been validated in preclinical and clinical settings, the PW remains a conceptual pharmacodynamic construct at this stage; it has not yet been experimentally defined or prospectively validated as introduced here and should be interpreted as a hypothesis-generating framework rather than an established pharmacological parameter.

Unlike the MSW, which is largely concentration-based, the PW emerges from the interaction among antimicrobial exposure, bacterial physiological state, and infection-site conditions. Consequently, it should not be viewed as a fixed concentration range but as a dynamic pharmacological zone influenced by bacterial dormancy, biofilm architecture, intracellular localization, tissue penetration, and local microenvironmental conditions [12,15,31,50].

The relationship between the PW and the MSW may have important evolutionary implications. By prolonging bacterial survival during therapy, persister populations create opportunities for repeated cycles of antimicrobial exposure, recovery, and re-exposure. This extended survival period increases the probability that resistant mutants will emerge and become established. Experimental studies by Windels et al. demonstrated that persistence can accelerate the evolution of resistance by extending the time available for adaptive mutations to arise [28]. Similarly, mechanistic analyses by Eisenreich et al. highlighted the interplay among persistence, stress-response activation, DNA repair pathways, and mutagenesis processes that may facilitate progression from phenotypic survival to stable genetic resistance [29].

These observations support a conceptual model in which persistence and resistance represent interconnected stages within a broader continuum of antimicrobial failure [14,29]. In this framework, bacterial populations may first enter the PW, where phenotypic survival predominates, before progressing to conditions that favor the selection of resistance within the MSW.

Traditional PK/PD optimization seeks to maximize bacterial killing while minimizing exposure within the MSW [44,45]. A persistence-oriented approach would additionally seek to minimize exposure conditions that permit persister survival and subsequent regrowth. This objective aligns closely with the concept of PPE, which focuses on preventing bacterial recovery after treatment cessation rather than solely achieving bacterial killing during therapy.

Although the PW remains a conceptual framework requiring experimental validation, it provides a useful extension of classical PK/PD theory by integrating persister biology into antimicrobial pharmacology. Together, the PW and PPE frameworks encourage a shift from evaluating short-term microbiological response alone toward achieving durable bacterial eradication and preventing persistence-driven relapse [6,14].

## 7. Target-Site Pharmacology and Biofilm Sanctuaries

Plasma pharmacokinetic parameters remain the most important way to determine how much of an antimicrobial to administer, but they are not always the best way to assess how much of the drug reaches the target site [31]. This limitation becomes particularly relevant in the context of bacterial persistence, where surviving subpopulations are frequently located within anatomical and physiological niches characterized by restricted antibiotic penetration, altered bacterial physiology, and unfavorable microenvironmental conditions [7,14,50].

Bacterial populations residing within abscesses, necrotic tissues, prosthetic device infections, bone compartments, pulmonary mucus, intracellular niches, or mature biofilms may encounter drug concentrations substantially lower than those measured in the systemic circulation [12,31,71]. As a result, apparently adequate plasma PK/PD target attainment may coexist with insufficient antimicrobial activity at the site of infection [31,44].

This discrepancy contributes to treatment failure, relapse, and chronic infection despite adherence to guideline-recommended antibiotic regimens [7,14].

The concept of target-site pharmacology is therefore central to understanding persistence. Effective eradication requires not only adequate systemic exposure but also sufficient antibiotic penetration, retention, and activity within the microenvironments that harbor dormant bacterial populations. Importantly, local antibiotic activity is influenced not only by drug concentration but also by environmental factors such as pH, oxygen availability, nutrient gradients, host immune activity, protein binding, and tissue architecture [15,31,50]. These variables can profoundly alter both bacterial physiology and antibiotic efficacy.

Many chronic infections contain what may be considered pharmacological sanctuaries, localized environments in which antimicrobial exposure is reduced, and bacterial survival is favored. Examples include avascular necrotic tissue, infected prosthetic materials, osteomyelitic lesions, cystic fibrosis airways, chronic wounds, endocardial vegetations, and intracellular compartments within phagocytic cells [7,31,33,50,71]. Within these sanctuaries, bacteria frequently adopt slow-growing or dormant phenotypes that further reduce susceptibility to antibiotic killing [6,8,15].

Biofilms are among the most clinically relevant pharmacological sanctuaries. Biofilms are highly organized multicellular bacterial communities embedded within a self-produced extracellular polymeric matrix composed of polysaccharides, proteins, extracellular DNA, and host-derived components [12,32]. These structures are encountered in a wide range of clinical settings, including chronic respiratory infections, urinary tract infections, prosthetic joint infections, cardiovascular device infections, chronic wounds, and catheter-associated infections [12,32]. The remarkable resilience of biofilms cannot be explained solely by impaired antibiotic diffusion. Although the extracellular matrix may delay or reduce penetration of certain antimicrobial agents, biofilm-associated tolerance and persistence primarily arise from profound physiological heterogeneity within the bacterial community [12,33]. Cells located in different biofilm regions experience distinct microenvironmental conditions, generating spatial gradients of oxygen tension, nutrient availability, pH, metabolic activity, and waste accumulation [12,32]. As oxygen and nutrients become depleted in deeper biofilm layers, bacterial cells progressively shift toward slow growth or dormancy. Because many bactericidal antibiotics depend on active cellular processes, including cell-wall synthesis, DNA replication, transcription, and protein synthesis, reduced metabolic activity markedly decreases antibiotic-mediated killing. Consequently, biofilms become highly enriched in tolerant and persister populations that survive exposures that eliminate their planktonic counterparts [8,12,15,33].

Recent work by Yan and Bassler has highlighted how bacterial communities exploit collective survival mechanisms to withstand antibiotic exposure, emphasizing the central role of persistence and phenotypic heterogeneity in biofilm resilience [12]. Similarly, Soares and colleagues reviewed the mechanisms of persistence and tolerance in *Pseudomonas aeruginosa* biofilms and highlighted that conventional antimicrobial regimens often fail to eradicate persister reservoirs despite prolonged or combination-based treatments [33]. These observations reinforce the concept that biofilm-associated infections should be viewed not merely as infections with impaired antibiotic penetration but as dynamic ecological systems that actively promote persister formation. Intracellular bacterial persistence represents another major therapeutic challenge. Pathogens such as *Staphylococcus aureus*, *Salmonella enterica*, *Listeria monocytogenes*, and *Mycobacterium tuberculosis* can persist within host cells, where antibiotic access may be limited and bacterial metabolic activity profoundly altered [50,72].

Intracellular compartments often exhibit acidic pH, oxidative stress, nutrient restriction, and reduced oxygen availability, conditions that further favor dormancy and phenotypic adaptation [50]. Consequently, antibiotic efficacy depends not only on systemic exposure but also on the drug’s ability to accumulate within the relevant intracellular compartment and remain active under local conditions [19,72].

These considerations have important implications for antimicrobial development. Traditional susceptibility testing is generally performed under standardized laboratory conditions that fail to replicate the complexity of microenvironments at the infection site [6,13]. As a result, susceptibility profiles may overestimate clinical efficacy in infections dominated by biofilm-associated, intracellular, or deeply dormant bacterial populations [14,50]. A significant translational limitation in target-site pharmacology is that existing techniques do not directly quantify the free antibiotic concentrations that bacteria encounter in complex infection environments. Microdialysis is an effective method for evaluating tissue pharmacokinetics because it enables repeated measurements of unbound extracellular drug concentrations within defined interstitial compartments [31,73,74]. However, microdialysis remains an approximation of infection-site exposure rather than a direct measurement of antibacterial target engagement. Probe placement, calibration and recovery procedures, tissue trauma caused by catheter insertion, local perfusion, protein binding, inflammation, edema, necrosis, and spatial heterogeneity may all influence measured concentrations [31,73,74,75]. Moreover, microdialysis generally samples extracellular interstitial fluid and therefore cannot reliably quantify intracellular antibiotic exposure, concentrations within abscess cores, necrotic tissue, granulomas, endocardial vegetations, or deep biofilm layers [31,73,76].

Contemporary molecular imaging, nuclear imaging, fluorescence-based methods, and advanced microscopy provide valuable data on infection localization, tissue distribution, and, in certain experimental contexts, antibiotic penetration. However, these techniques have significant limitations. Most imaging modalities do not quantify the pharmacologically active unbound drug fraction, frequently lack adequate spatial and temporal resolution to resolve subcellular or sub-biofilm gradients, and may not differentiate between total drug accumulation and microbiologically effective exposure [77,78]. In mature biofilms, gradients of oxygen, pH, nutrients, redox potential, and matrix composition generate microdomains where antibiotic penetration, stability, uptake, and activity can differ significantly from those in surrounding tissues [12,79]. Therefore, plasma PK/PD target attainment, measured tissue concentrations, and imaging-derived drug distribution represent complementary yet indirect indicators of antimicrobial exposure. To date, none of these methods provides routine, standardized measurement of the actual local, intracellular, or sub-biofilm-free antibiotic concentrations that persister cells encounter. This methodological limitation underscores the need to integrate target-site PK, dynamic infection models, biofilm-specific assays, and pharmacodynamic regrowth endpoints to develop persistence-oriented dosing strategies. Future anti-persister strategies will therefore require a more comprehensive integration of microbiology with tissue pharmacology, spatial drug distribution, and infection-site biology.

Several approaches are being explored to improve delivery to persister reservoirs. These include antimicrobial coatings for implanted devices, biofilm-disrupting agents, bacteriophage-based therapies, enzyme-mediated matrix degradation, targeted drug-delivery systems, liposomal formulations, nanoparticles, stimuli-responsive biomaterials, and other nanotherapeutic platforms designed to enhance local antibiotic concentrations while simultaneously disrupting protective bacterial microenvironments [35,80,81].

**Figure 4:**
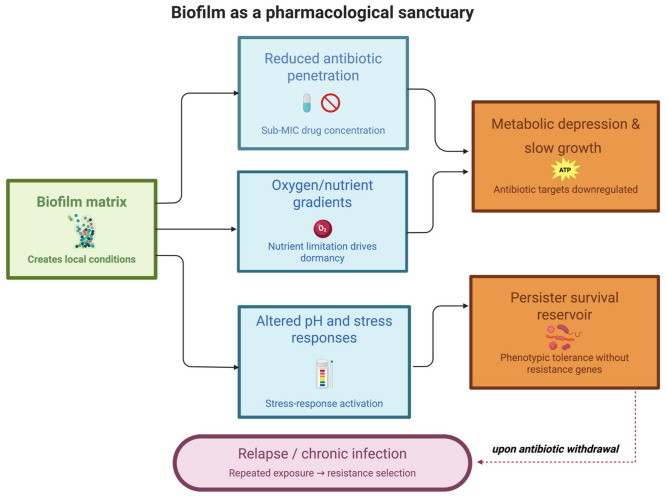
Biofilm as a pharmacological sanctuary. The biofilm matrix creates a physicochemical microenvironment that drives antibiotic tolerance through three converging mechanisms: reduced drug penetration, oxygen/nutrient gradients that impose metabolic restriction, and altered pH that activates stress responses. These conditions sustain a persister survival reservoir of viable, non-replicating cells that escape killing without genetic resistance. Upon antibiotic withdrawal, persisters resume replication, driving relapse and iterative resistance selection. Arrows indicate forward causal flow; the dashed coral arrow denotes the relapse pathway upon antibiotic withdrawal. Color coding: green = primary cause (biofilm matrix); blue = mechanistic nodes (microenvironmental conditions); orange = downstream effects (metabolic depression, persister reservoir); coral = clinical outcome (relapse/chronic infection). MIC, minimum inhibitory concentration. Figure created with BioRender (https://www.biorender.com, accessed on 22 June 2026).

Understanding target-site pharmacology and the formation of biofilm sanctuaries is therefore essential for developing therapeutic strategies capable of achieving not only microbiological suppression but durable bacterial eradication (Figure 4).

## 8. TDM as a Tool for Persistence Prevention

TDM has become an increasingly important component of modern antimicrobial stewardship, particularly in critically ill patients, individuals with altered pharmacokinetics, and infections requiring prolonged treatment or exposure to potentially toxic antibiotics [44]. Traditionally, TDM has been used to optimize efficacy while minimizing toxicity by ensuring achievement of established PK/PD targets such as fT > MIC, AUC/MIC, or Cmax/MIC. This approach has significantly improved the management of severe infections and has become standard practice for several antimicrobial agents, including vancomycin, aminoglycosides, and selected β-lactams [44,47,70].

However, the emergence of persistence as a clinically relevant determinant of treatment failure suggests that conventional TDM objectives may be incomplete. Achievement of traditional PK/PD targets does not necessarily guarantee eradication of dormant bacterial subpopulations, particularly in complex infections characterized by biofilm formation, intracellular persistence, impaired tissue penetration, or heterogeneous bacterial physiology. Consequently, antibiotic exposures considered optimal by current standards may still allow persister survival and subsequent relapse [7,12,14,31,50].

From a persistence-oriented perspective, the goal of TDM extends beyond achieving bacterial killing during therapy. Instead, TDM may be viewed as a tool for minimizing the probability of persister survival by reducing exposure conditions that favor entry into or maintenance within the PW. In this framework, the objective is not merely to reach a predefined PK/PD threshold but to ensure that antibiotic exposure remains sufficient, sustained, and appropriately distributed to prevent post-treatment bacterial recovery. This concept introduces the possibility of precision anti-persister dosing, in which antimicrobial therapy is individualized according to both patient-specific pharmacokinetics and infection-specific pharmacodynamic challenges [44,47,70].

Rather than relying exclusively on plasma concentrations, dosing strategies would aim to optimize exposure at the actual site of infection while accounting for factors known to promote persistence, including biofilm formation, intracellular localization, tissue necrosis, altered perfusion, and bacterial dormancy [31,50,82]. Importantly, a persistence-oriented dosing strategy should not be interpreted as simply administering higher antibiotic doses or prolonging treatment duration. Escalating exposure indiscriminately may increase toxicity without necessarily improving eradication of dormant cells, particularly when antibiotic activity is limited by reduced target engagement rather than insufficient concentration [8,14,15]. Instead, dosing interventions should be mechanism-based, pathogen-specific, and tailored to the pharmacological characteristics of both the antibiotic and the site of infection.

Several practical approaches may help minimize persistence by optimizing antimicrobial exposure. Early attainment of therapeutic concentrations is particularly important because delayed achievement of target concentrations may allow bacterial adaptation and persister formation during the initial phase of therapy [44,50]. In selected clinical scenarios, appropriate loading doses can accelerate attainment of effective exposure and reduce the time spent within potentially persistence-promoting concentration ranges. Similarly, prolonged or continuous infusion strategies for β-lactam antibiotics may enhance bacterial killing by maintaining drug concentrations above relevant pharmacodynamic thresholds throughout the dosing interval [46]. Such approaches may be especially valuable in critically ill patients exhibiting augmented renal clearance or significant pharmacokinetic variability, conditions that frequently lead to suboptimal exposure despite standard dosing regimens [44].

For antibiotics such as vancomycin, AUC-guided monitoring represents another example of precision dosing that may help optimize antimicrobial exposure while reducing toxicity [70]. More broadly, Bayesian forecasting and model-informed precision dosing are increasingly enabling real-time adjustment of antimicrobial regimens according to individual patient characteristics, offering opportunities to refine treatment beyond traditional population-based dosing approaches [47,83].

A critical evolution in future TDM strategies may involve incorporating target-site pharmacology into routine clinical decision-making. Plasma concentrations provide only indirect information regarding exposure within infected tissues, where bacterial populations reside [31,82]. The discrepancy between systemic and local antibiotic exposure can be particularly pronounced in abscesses, osteomyelitis, prosthetic device infections, pulmonary biofilms, and intracellular infections [31,50,71]. Therefore, an exclusive focus on plasma PK/PD targets may overlook pharmacological conditions that favor persister survival within these protected niches.

Emerging technologies, including microdialysis-based tissue pharmacokinetic studies, physiologically based pharmacokinetic (PBPK) modeling, and advanced imaging techniques, may eventually allow more accurate estimation of antibiotic exposure at infection sites [31,73,82]. Such approaches could facilitate the identification of exposure profiles associated not only with bacterial killing but also with the suppression of persistence and the prevention of relapse [6,14].

The integration of TDM with source-control strategies represents another critical component of persistence-oriented therapy. Surgical drainage, removal of infected devices, debridement of necrotic tissue, and disruption of biofilms can dramatically alter local pharmacokinetics and reduce the burden of protected bacterial populations [12,71,84]. Consequently, optimal anti-persister therapy should be viewed as the result of coordinated pharmacological and procedural interventions rather than antibiotic exposure alone.

Ultimately, the future role of TDM may evolve from conventional target attainment toward a broader precision-medicine framework focused on durable bacterial eradication. Within this paradigm, therapeutic monitoring would aim not only to ensure adequate drug exposure but also to minimize persistence-promoting conditions, reduce the likelihood of post-treatment regrowth, and prevent relapse. Such an approach aligns closely with the concepts of PPE and the PW, providing a practical clinical strategy for translating persister biology into individualized antimicrobial therapy. Although prospective validation is still required, the integration of TDM, target-site pharmacology, and precision dosing represents one of the most promising avenues for transforming persistence from an unavoidable cause of treatment failure into a modifiable pharmacological target [14,44,47].

## 9. Combination and Sequential Therapy

Combination therapy may be pharmacologically justified when it expands physiological target coverage across bacterial populations occupying different metabolic states, anatomical compartments, or microenvironmental niches. Unlike conventional monotherapy, which is often optimized against actively replicating bacteria, combination regimens can simultaneously target growing cells, dormant persisters, intracellular reservoirs, and biofilm-associated populations [14,34,36]. In this context, the rationale for combination therapy extends beyond spectrum broadening or resistance prevention and becomes a strategy for overcoming bacterial physiological heterogeneity. As highlighted by Defraine and colleagues, successful eradication of persister populations may require therapeutic approaches that go beyond standard antibiotic monotherapy and specifically address the biological mechanisms underlying persistence [36]. The effectiveness of combination therapy may derive from several complementary pharmacological mechanisms, including enhanced bacterial killing, improved penetration into protected niches, disruption of biofilm architecture, metabolic activation of dormant cells, and prevention of bacterial regrowth following antibiotic withdrawal [11,22,84].

Several clinically relevant combinations illustrate these principles. β-lactam–aminoglycoside combinations may exploit cell-wall disruption to facilitate aminoglycoside uptake, thereby enhancing bactericidal activity in selected pathogens and clinical contexts [85]. Daptomycin combined with β-lactams has demonstrated synergistic activity against difficult-to-treat Gram-positive infections, partly through β-lactam-mediated alterations in bacterial membrane physiology that increase daptomycin binding and activity [86,87,88]. Rifampicin-containing regimens remain valuable in selected prosthetic-device and implant-associated staphylococcal infections because of rifampicin’s ability to penetrate biofilms and target sessile bacterial populations, although careful use is required to prevent the rapid emergence of resistance [71].

Other combinations have been specifically explored to address persistence-related mechanisms. Fosfomycin-containing regimens may improve activity against biofilm-associated pathogens and multidrug-resistant organisms through their unique mechanism of action and favorable tissue penetration [89,90,91].

Likewise, combinations of conventional antibiotics with metabolic adjuvants have emerged as a promising strategy to enhance the susceptibility of dormant bacterial populations. By stimulating bacterial metabolism and promoting exit from quiescent states, these approaches may increase the efficacy of antibiotics whose activity depends on active cellular processes [15,22].

The integration of anti-biofilm therapies with antimicrobial treatment represents another important area of development. Biofilm-disrupting agents, matrix-degrading enzymes, quorum-sensing inhibitors, and compounds targeting extracellular polymeric substances may enhance antibiotic access to protected bacterial communities and reduce the survival of persister reservoirs [12,35,84]. Similarly, increasing attention has focused on phage–antibiotic combinations, which may provide complementary mechanisms of bacterial killing and biofilm disruption while potentially reducing the likelihood of persistence-driven relapse [92,93,94].

From a pharmacological standpoint, the success of combination therapy depends not only on antimicrobial potency but also on the degree of mechanistic complementarity between the selected agents. Combinations that target distinct physiological pathways, bacterial states, or anatomical compartments may provide greater benefits than regimens involving drugs with overlapping mechanisms of action [11,36]. This concept is particularly relevant in chronic infections characterized by pronounced bacterial heterogeneity and complex tissue microenvironments.

Nevertheless, combination therapy should not be viewed as a universal solution to persistence. The addition of multiple agents may increase the risk of toxicity, drug–drug interactions, treatment complexity, disruption of the host microbiota, and ecological selection pressure favoring antimicrobial resistance. Furthermore, not all combinations are synergistic; antagonistic interactions may occur when one agent suppresses the bacterial metabolic activity required for another antibiotic to exert its bactericidal effect [11,93]. Consequently, combination regimens should be selected based on a clear pharmacological rationale, considering pathogen biology, infection-site characteristics, target-site drug exposure, bacterial physiological state, and available clinical evidence.

Future precision antimicrobial strategies may increasingly rely on mechanism-based combinations specifically designed to prevent persister survival, minimize time spent within the PW, and achieve PPE. In this framework, the ultimate objective is not simply enhanced bacterial killing during therapy but the durable eradication of bacterial populations that can drive relapse and the evolution of subsequent resistance [14,28,29] (Table 4).

## 10. Emerging Pharmacological Strategies for Persistence Prevention

### 10.1. Metabolic Activation

Among the emerging strategies to overcome bacterial persistence, metabolic activation is one of the most conceptually innovative and pharmacologically attractive approaches [15,22,30]. Unlike conventional antimicrobial optimization, which primarily seeks to increase antibiotic exposure, metabolic activation focuses on modifying bacterial physiology to restore susceptibility to existing antimicrobial agents. The rationale for this approach stems from the observation that many bactericidal antibiotics depend on active bacterial metabolism to exert their lethal effects [8,30]. Processes such as cell-wall synthesis, DNA replication, transcription, protein synthesis, membrane energization, and reactive oxygen species generation are central to antibiotic activity. When bacteria enter metabolically quiescent states, these pathways become downregulated, substantially reducing antibiotic effectiveness despite preserved genetic susceptibility [6,8,13]. Consequently, persister survival often reflects a physiological absence of target engagement rather than true antimicrobial resistance.

From a pharmacological perspective, persistence can therefore be interpreted as a state of transient metabolic uncoupling between antibiotic exposure and bacterial killing. Metabolic activation seeks to reverse this uncoupling by stimulating cellular processes required for antibiotic action, thereby transforming dormant persisters into pharmacologically accessible targets. One of the seminal demonstrations of this principle was provided by Allison and colleagues, who showed that specific metabolites could dramatically potentiate aminoglycoside-mediated killing of bacterial persisters. In their study, supplementation with metabolites such as mannitol, fructose, and glucose enhanced proton motive force generation, promoted aminoglycoside uptake, and restored antibiotic susceptibility in otherwise tolerant bacterial populations [22]. Importantly, these metabolites possessed little direct antibacterial activity on their own. Their therapeutic value derives from their ability to alter bacterial physiology, thereby increasing antibiotic efficacy.

Rather than developing entirely new antibiotics, this strategy aims to exploit bacterial metabolic plasticity to improve the performance of currently available drugs [15,22,95]. Subsequent investigations have expanded this concept beyond aminoglycosides. Multiple metabolic pathways have been implicated in persister formation and survival, including ATP homeostasis, central carbon metabolism, the tricarboxylic acid cycle, respiratory activity, redox balance, and stress-response signaling [15,30,96,97]. Pharmacological manipulation of these pathways may influence the depth of dormancy and determine whether bacterial populations remain protected or become susceptible to antibiotic killing.

Numerous studies have demonstrated that low intracellular ATP levels correlate with increased persister formation, whereas restoration of metabolic activity may enhance antibiotic susceptibility [20,34,98]. These observations suggest that bacterial energetics may represent a key pharmacological target for future anti-persister interventions. In this framework, metabolic activation may be viewed as a strategy to reduce dormancy depth and shift bacterial populations toward physiological states that support antibiotic-mediated killing.

The concept also aligns closely with the broader notion of wake-up therapy, in which dormant bacterial cells are induced to re-enter metabolically active states before or during antibiotic exposure [19,99]. Although the terms are sometimes used interchangeably, metabolic activation may be considered the mechanistic basis underlying many wake-up strategies. By stimulating bacterial respiration, ATP production, nutrient utilization, or biosynthetic activity, these interventions seek to convert phenotypic tolerance into pharmacological vulnerability. Importantly, metabolic activation does not necessarily require complete restoration of bacterial growth. Even partial reactivation of key metabolic pathways may be sufficient to enhance antibiotic uptake, improve target engagement, or increase susceptibility to bactericidal mechanisms [21,22]. This distinction is clinically relevant because excessive stimulation of bacterial proliferation could theoretically increase bacterial burden or exacerbate infection. The ideal anti-persister strategy would therefore selectively restore antibiotic-sensitive physiological functions without promoting uncontrolled bacterial expansion.

The therapeutic implications of metabolic activation extend beyond planktonic bacteria. Biofilm-associated populations, intracellular reservoirs, and nutrient-limited bacterial communities frequently display profound metabolic heterogeneity and represent major sources of persister cells [12,33,50]. Consequently, metabolic adjuvants capable of reprogramming bacterial physiology within these protected environments may significantly enhance the efficacy of conventional antibiotic regimens.

Despite its promise, several challenges remain before metabolic activation can be routinely translated into clinical practice. The metabolic requirements of persister populations vary among bacterial species, infection models, and microenvironmental conditions [15,34]. Furthermore, optimal combinations of metabolic adjuvants and antibiotics have yet to be established for most clinically relevant pathogens. Future studies integrating systems biology, metabolomics, dynamic infection models, and target-site pharmacology will be essential to identify the most effective metabolic interventions [30,31,50].

Rather than attempting to overcome persistence through escalating antibiotic exposure alone, it seeks to restore the biological conditions necessary for antibiotic efficacy. In this sense, metabolic activation transforms persister cells from inaccessible survivors into actionable pharmacological targets and may become a key component of future precision strategies to achieve PPE and durable bacterial eradication.

### 10.2. Wake-Up Therapy

Wake-up therapy is one of the most intriguing emerging strategies for eradicating bacterial persister cells [34,54,99]. Metabolic activation aims to improve cellular energetics and restore antibiotic susceptibility by stimulating bacterial metabolism. In contrast, wake-up therapy directly targets the dormant physiological state that causes persistence. The fundamental objective is to induce persister cells to exit quiescence and re-enter metabolically active states in which conventional bactericidal antibiotics can effectively exert their lethal activity [100,101,102].

The rationale for this approach derives from the observation that persistence is not a permanent bacterial trait but rather a reversible physiological condition [6,51]. Persister cells remain viable because they transiently suppress cellular processes that are targeted by most antibiotics, including DNA replication, protein synthesis, cell-wall turnover, and energy metabolism [8,13].

Consequently, antibiotics often fail not because their targets are genetically altered, but because those targets become physiologically inactive. Wake-up therapy seeks to reverse this state of reduced target engagement by forcing dormant bacteria to resume biological functions necessary for antibiotic-mediated killing.

From a pharmacological perspective, wake-up therapy can be viewed as an attempt to reduce the depth of dormancy, a concept increasingly recognized as a critical determinant of persister survival [53,103]. Persister populations are not physiologically homogeneous; rather, they occupy a spectrum ranging from shallow dormancy, in which cells can rapidly resume growth, to deeply dormant states approaching viable-but-non-culturable (VBNC) phenotypes [25,53]. The depth of dormancy influences both the likelihood of spontaneous resuscitation and the responsiveness to therapeutic interventions [100,101,103]. Wake-up strategies aim to shift bacterial populations toward more metabolically active states that are susceptible to antimicrobial attack.

Several molecular pathways involved in persister formation and maintenance have emerged as potential therapeutic targets. Among the most extensively studied are toxin–antitoxin systems, which have been implicated in growth arrest, stress adaptation, and persistence, although their contributions may vary substantially across bacterial species, genetic backgrounds, and experimental conditions [54,102,104]. Pharmacological manipulation of these systems may facilitate the transition from dormancy to active growth, thereby restoring susceptibility to antibiotic killing.

The stringent response represents another attractive target. This highly conserved bacterial stress-adaptation mechanism is largely mediated by the alarmone guanosine tetraphosphate and pentaphosphate, more commonly referred to as (p)ppGpp, which orchestrate widespread transcriptional and metabolic reprogramming during nutrient limitation and environmental stress. Elevated (p)ppGpp levels promote growth arrest, stress tolerance, and persister formation [105,106]. Consequently, inhibition of stringent-response signaling has been proposed as a strategy to prevent or reverse persistence [54,105].

Additional regulatory networks implicated in dormancy control include cyclic AMP (cAMP), cyclic di-GMP (c-di-GMP), quorum-sensing pathways, ribosomal hibernation mechanisms, and global stress-response regulators [12,107,108]. These interconnected signaling systems govern bacterial decisions regarding growth, biofilm formation, stress adaptation, and metabolic activity. Manipulating such pathways may allow controlled resuscitation of dormant populations and increase their vulnerability to antimicrobial therapy.

Small molecules that modulate intracellular second messengers or disrupt dormancy-maintaining circuits may provide a means to synchronize bacterial resuscitation prior to antibiotic administration [105,106]. In theory, such synchronization could transform heterogeneous persister populations into more uniform and pharmacologically vulnerable targets. However, wake-up therapy also presents unique challenges and potential risks. Reactivating dormant bacterial populations in the absence of adequate antimicrobial coverage could paradoxically promote bacterial proliferation, increase pathogen burden, and exacerbate infection. For this reason, wake-up interventions should not be viewed as standalone therapies but rather as components of carefully coordinated treatment strategies. Their success depends on precise temporal coupling with effective bactericidal exposure that eliminates reactivated cells before substantial bacterial expansion occurs [54,99,109].

This requirement introduces an important pharmacological principle: resuscitation must be synchronized with PPE. In practical terms, the therapeutic window between bacterial awakening and antibiotic killing may be narrow, necessitating careful optimization of antibiotic timing, concentration, and target-site exposure. The effectiveness of wake-up therapy may therefore depend as much on antimicrobial pharmacokinetics and pharmacodynamics as on the resuscitation stimulus itself [31,44].

The complexity of these approaches has been highlighted in recent reviews that emphasized both the considerable therapeutic potential and the substantial translational challenges associated with persister resuscitation strategies [50,54,99,109]. Although numerous molecular targets have been identified in experimental systems, translating these findings into clinically applicable therapies remains difficult because dormancy mechanisms vary across bacterial species, infection models, and micro-environmental conditions [15].

Wake-up therapy therefore seeks to restore susceptibility to existing antibiotics by manipulating bacterial physiology. By converting phenotypically protected cells into pharmacologically accessible targets, wake-up strategies may ultimately complement metabolic activation, anti-biofilm therapies, and precision dosing approaches within an integrated anti-persister treatment paradigm. As our understanding of bacterial dormancy deepens, the combination of controlled resuscitation with optimized antimicrobial exposure may become a key strategy for minimizing persistence, reducing relapse, and achieving durable bacterial eradication.

### 10.3. Direct Persister-Targeting Molecules and Biomaterials

While metabolic activation and wake-up therapies seek to restore susceptibility of dormant bacterial populations to conventional antibiotics, an alternative and increasingly attractive strategy is the direct targeting of persister cells themselves [14,34,36]. The fundamental premise of this approach is that persisters, despite their profound physiological adaptations, retain specific vulnerabilities that can be exploited therapeutically. Rather than forcing bacteria to resume growth before treatment, direct anti-persister agents aim to eliminate dormant cells irrespective of their metabolic state. Most currently available antibiotics rely on active cellular processes, including cell-wall synthesis, DNA replication, transcription, translation, or energy metabolism, to exert their bactericidal effects [8,13,30]. Consequently, dormant persister cells often evade killing because these biological pathways are temporarily suppressed. Direct persister-targeting agents seek to bypass this dependence on bacterial growth by attacking cellular functions that remain essential even during dormancy.

Among the most extensively studied examples are compounds that target bacterial proteostasis. A landmark discovery in this field was the demonstration that activation of the bacterial caseinolytic protease P (ClpP) can induce catastrophic intracellular protein degradation and eliminate persister populations. Conlon and colleagues showed that acyldepsipeptide (ADEP) antibiotics activate ClpP independently of its normal regulatory mechanisms, thereby triggering uncontrolled proteolysis and eradicating persistent Staphylococcus aureus populations in experimental chronic biofilm infections. Importantly, this killing mechanism remains active even in non-dividing bacterial cells, highlighting the potential of proteostasis disruption as a growth-independent therapeutic strategy [20].

The success of ClpP-targeting approaches has stimulated broader interest in the bacterial protein-quality-control machinery as a pharmacological target. Chaperone systems, protein-folding pathways, stress-response networks, and proteolytic complexes are increasingly recognized as critical determinants of persister survival. Because maintenance of proteome integrity remains necessary even during dormancy, disruption of these systems may represent a particularly effective means of eliminating persistent bacterial populations [20,34].

Membrane-active compounds constitute another promising class of direct anti-persister agents. Unlike many conventional antibiotics, membrane-targeting molecules often exert bactericidal activity independently of active bacterial growth. By disrupting membrane integrity, collapsing membrane potential, altering permeability, or interfering with essential membrane-associated functions, these agents can rapidly kill both actively growing and dormant bacteria [34,36,110]. The clinical success of compounds such as daptomycin has further stimulated interest in developing next-generation membrane-active therapeutics with potential activity against dormant or biofilm-associated bacterial population [14,36]. Similarly, antimicrobial peptides (AMPs) have attracted considerable attention because of their broad-spectrum activity and capacity to target bacterial membranes through mechanisms less dependent on metabolic state [110,111]. Many AMPs combine direct bactericidal activity with immunomodulatory effects, potentially providing dual benefits in the treatment of persistent infections. Their ability to penetrate biofilms and disrupt bacterial membranes makes them particularly attractive candidates for targeting persister reservoirs.

Beyond proteostasis and membrane disruption, several emerging compounds aim to exploit other growth-independent vulnerabilities. These include agents targeting oxidative-stress defense systems, energy maintenance pathways, DNA integrity, protein aggregation processes, and stress-response regulators [15,34,56]. Collectively, these approaches reflect a shift from targeting bacterial proliferation to targeting the fundamental survival mechanisms that enable persistence.

The development of direct anti-persister therapies has also benefited from advances in nanotechnology and biomaterials science. One of the major challenges in persistence eradication is not only identifying effective agents but also ensuring adequate delivery to the protected niches where persister populations reside. Biofilms, intracellular compartments, prosthetic-device surfaces, necrotic tissues, and poorly vascularized infection sites frequently limit antibiotic penetration and create pharmacological sanctuaries that support bacterial survival [12,31,50]. Nanoparticle-based delivery systems offer several potential advantages in this context. By enhancing drug stability, improving tissue penetration, facilitating intracellular delivery, and enabling controlled drug release, nanoparticles may increase local exposure within persister-rich environments [81,112,113]. Furthermore, engineered nanomaterials can be designed to respond to specific environmental stimuli, such as pH, enzymatic activity, oxidative stress, or bacterial metabolites, thereby enabling targeted release of antimicrobial payloads directly at the infection site [35,112,113].

Advanced biomaterial-based platforms are also being explored as tools for the eradication of persistence. Antimicrobial coatings, drug-eluting implants, hydrogels, responsive polymers, and biofilm-disrupting biomaterials may provide sustained local delivery of anti-persister agents while simultaneously reducing bacterial colonization of medical devices [35]. Such approaches are particularly attractive for prosthetic joint infections, vascular device infections, chronic wounds, and other settings in which biofilm-associated persistence plays a dominant role [35,71]. Importantly, these technologies should not be viewed solely as delivery systems. Many biomaterials possess intrinsic anti-biofilm properties, can interfere with bacterial adhesion, or may alter local microenvironmental conditions that favor persistence. Consequently, biomaterial-based approaches increasingly represent active therapeutic interventions rather than passive drug carriers [35].

Despite considerable progress, most direct anti-persister agents remain at the preclinical or early translational stage. Challenges include selective toxicity, optimization of target specificity, avoidance of resistance development, and demonstration of efficacy in complex in vivo infection models [14,36,112]. Nevertheless, the field is rapidly evolving and reflects a fundamental change in antimicrobial drug discovery. Instead of focusing exclusively on bacterial growth inhibition, researchers are increasingly targeting the biological mechanisms that enable long-term survival under antibiotic stress. From a pharmacological standpoint, direct persister-targeting agents may constitute the most straightforward pathway to attain PPE. By eliminating bacterial populations that survive conventional antimicrobial therapy, these strategies have the potential to reduce relapse, shorten treatment duration, improve source-control outcomes, and ultimately transform persistence from a major cause of therapeutic failure into a tractable pharmacological target.

### 10.4. Host-Directed Therapy: Targeting the Host–Pathogen Interface

The majority of anti-persister strategies developed to date focus on directly targeting bacterial cells through enhanced antimicrobial exposure, metabolic activation, reversal of dormancy, or growth-independent killing mechanisms. However, increasing evidence suggests that persistence is not solely a bacterial phenomenon. Rather, it emerges from a dynamic interaction between the pathogen, the antimicrobial agent, and the host microenvironment [37,50]. In this view, persister formation and survival are shaped not only by bacterial physiology but also by host-derived signals, immune pressures, tissue conditions, and metabolic constraints [15,50].

This perspective has given rise to growing interest in host-directed therapies (HDTs) as complementary approaches for combating persistent infections. Instead of directly attacking bacterial cells, host-directed interventions seek to modify the biological environment in which persistence develops, thereby enhancing pathogen clearance while potentially reducing the selective pressures associated with conventional antibiotic therapy [37,114]. The rationale for host-directed therapy is supported by evidence that many host-associated stressors can promote bacterial tolerance and persistence. Exposure to oxidative stress, nutrient limitation, hypoxia, inflammatory mediators, intracellular confinement, metal sequestration, and immune-cell attack may induce bacterial stress-response pathways that favor dormancy and phenotypic adaptation [50]. Consequently, bacterial persistence can be viewed not only as a response to antibiotics but also as an adaptive reaction to host-imposed environmental pressures.

Among the most extensively investigated host-directed approaches is the modulation of macrophage function. Macrophages play a central role in controlling intracellular pathogens but may also provide protected niches in which persistent bacteria survive antibiotic exposure [50,115]. Enhancing macrophage antimicrobial activity through immunometabolic reprogramming, increased phagolysosomal maturation, improved production of reactive oxygen and nitrogen species, or optimized intracellular killing mechanisms may reduce the persistence of pathogens residing within host cells [19,115,116]. Such strategies may be particularly relevant for infections caused by *Mycobacterium tuberculosis*, *Salmonella enterica*, *Listeria monocytogenes*, and intracellular *Staphylococcus aureus* populations [19,50].

Autophagy modulation represents another promising avenue. Autophagy is a highly conserved cellular process involved in the degradation of intracellular pathogens, damaged organelles, and protein aggregates. Pharmacological enhancement of selective antibacterial autophagy may facilitate clearance of intracellular bacterial reservoirs that evade both immune defenses and antibiotic therapy [115,117,118]. Because autophagy influences cellular metabolism, inflammation, and pathogen elimination simultaneously, it represents an attractive multifunctional target in persistence-oriented therapy [119,120].

Cytokine-based interventions have also been explored as means of enhancing host defense mechanisms. Although systemic immune stimulation carries inherent risks, selective modulation of cytokine pathways may improve pathogen clearance in specific contexts [37,116]. The challenge lies in achieving sufficient enhancement of antimicrobial immunity without exacerbating tissue damage or pathological inflammation. Future approaches will likely focus on precision immunomodulation tailored to specific infection types and host immune profiles.

Particularly intriguing is the concept of trained immunity, whereby innate immune cells undergo long-lasting functional reprogramming following exposure to microbial or metabolic stimuli. Unlike classical adaptive immunity, trained immunity enhances the responsiveness of innate immune cells through epigenetic and metabolic remodeling [121,122]. By increasing the efficiency of pathogen recognition and clearance, trained immunity may reduce the probability that bacterial populations establish persistent reservoirs during infection [123]. Although clinical applications remain in early development, this field represents an emerging intersection between immunology, metabolism, and antimicrobial pharmacology [121,122].

Another area of growing interest is resolution pharmacology, which seeks not to amplify inflammation but to promote its controlled resolution. Persistent infections are frequently associated with chronic inflammatory states that simultaneously damage host tissues and create microenvironments favorable to bacterial persistence. Specialized pro-resolving mediators, lipid-derived signaling molecules, and other resolution-promoting agents may help restore tissue homeostasis, improve immune-cell function, and enhance bacterial clearance while limiting collateral tissue injury [124,125,126].

The host microbiome has also emerged as a potentially important determinant of persistence. Commensal microbial communities influence immune maturation, colonization resistance, nutrient availability, and inflammatory signaling [127,128]. Disruption of these communities through antibiotic exposure may inadvertently create ecological conditions that favor pathogen persistence and recurrent infection [128,129]. Consequently, microbiome-directed interventions, including probiotics, prebiotics, microbial metabolites, microbiota restoration strategies, and fecal microbiota transplantation in selected settings, are increasingly being explored as adjunctive approaches to infection management [127,128,130].

From a pharmacological perspective, host-directed therapies offer several theoretical advantages. Because they target host pathways rather than bacterial structures, they may impose less direct selective pressure for the development of resistance, although this advantage remains context-dependent and requires clinical validation [37,114]. Furthermore, they can potentially act synergistically with conventional antibiotics by modifying the infection microenvironment, improving immune-mediated bacterial clearance, and reducing the physiological conditions that favor persistence [19,37,50].

However, significant challenges remain. Host responses vary considerably among individuals, and excessive immune modulation may result in toxicity, immunopathology, or impaired host defense [37,131]. In addition, the complex interplay among immune responses, bacterial physiology, tissue pharmacology, and antimicrobial exposure remains incompletely understood. Consequently, the successful implementation of host-directed strategies will likely require a precision-medicine approach that integrates microbiological, immunological, and pharmacological information.

Ultimately, host-directed therapies represent a paradigm shift in anti-persister treatment. Rather than focusing exclusively on the pathogen, these approaches recognize persistence as an emergent property of the host–pathogen–drug interaction [37,50]. By modifying the biological environment that supports bacterial survival, host-directed interventions may complement conventional antimicrobial therapy and contribute to durable bacterial eradication, particularly in chronic, relapsing, biofilm-associated, and intracellular infections. As the field moves toward precision antimicrobial stewardship, integration of host-directed therapies with optimized antibiotic exposure, PPE, target-site pharmacology, and direct anti-persister agents may provide the most comprehensive strategy for preventing persistence-driven treatment failure and relapse.

### 10.5. Phage–Antibiotic Strategies: Exploiting Complementary Mechanisms Against Persistent Infections

Bacteriophage-based therapies have re-emerged as promising adjunctive approaches for the treatment of difficult-to-treat bacterial infections, particularly in the context of antimicrobial resistance, biofilm-associated disease, and chronic relapsing infections [38,39,132]. Although phage therapy was originally developed long before the antibiotic era, recent advances in molecular biology, genomics, and personalized microbiology have renewed interest in its clinical application as a complement to conventional antimicrobial treatment [38].

From a persistence-oriented perspective, the relevance of bacteriophages extends beyond their direct antibacterial activity. Phages possess several properties that may help overcome key biological and pharmacological mechanisms underlying persister survival. Their ability to replicate at sites of infection, penetrate biofilm structures, disrupt bacterial communities, and exert bactericidal activity through mechanisms distinct from conventional antibiotics makes them attractive candidates for integrated anti-persister strategies [38,94,132].

One of the most important potential advantages of phage therapy is its activity within biofilm-associated infections. Biofilms represent major reservoirs of persister cells and frequently act as pharmacological sanctuaries where antibiotic penetration is reduced and bacterial metabolic activity is profoundly altered [12,33,132]. Certain bacteriophages produce depolymerases and other enzymes that degrade extracellular polymeric matrices, thereby facilitating access to bacteria embedded within biofilms [132]. This activity may not only enhance phage-mediated killing but also improve antibiotic penetration into previously protected bacterial niches.

The combination of phages and antibiotics has attracted particular attention due to the potential for complementary or synergistic interactions. Several experimental studies have demonstrated that phage exposure can increase bacterial susceptibility to antibiotics by altering bacterial physiology, disrupting biofilm architecture, modifying membrane properties, or selecting bacterial variants with reduced fitness. Conversely, antibiotics may enhance phage efficacy by altering bacterial growth dynamics and increasing their accessibility to target populations. These reciprocal interactions have led to the concept of phage–antibiotic synergy (PAS), in which the combined effect exceeds that of either intervention alone [92,93,94].

From a pharmacological standpoint, phage–antibiotic combinations may provide broader physiological target coverage than conventional antimicrobial regimens. Whereas antibiotics frequently display reduced activity against dormant bacterial populations, phages may continue to exert antibacterial pressure within heterogeneous bacterial communities and biofilm structures, although their efficacy still depends on host range, bacterial receptor availability, local immune factors, and infection-site accessibility [38,94]. Consequently, phage-based approaches may help reduce the size of persister reservoirs that survive conventional antibiotic exposure and contribute to relapse [132].

Another attractive feature of bacteriophage therapy is its potential to address target-site limitations. Because phages can amplify locally in the presence of susceptible bacteria, they differ fundamentally from conventional antimicrobial agents, whose activity is largely constrained by systemic pharmacokinetics [38]. Although phage distribution is also influenced by anatomical and immunological factors, their capacity for local replication introduces a unique pharmacological dimension that may be particularly relevant in infections characterized by spatially heterogeneous bacterial populations.

Nevertheless, several challenges remain before phage therapy can be routinely integrated into persistence-oriented treatment strategies. Bacterial resistance to phages, narrow host specificity, manufacturing complexity, regulatory considerations, and variability in clinical protocols continue to limit widespread implementation [38]. Furthermore, most clinical studies have focused on treatment-refractory, multidrug-resistant, or biofilm-associated infections rather than on evaluating persistence-related endpoints such as post-treatment regrowth, relapse prevention, or reductions in persister populations.

Despite these limitations, the growing number of clinical investigations registered on ClinicalTrials.gov and the increasing number of compassionate-use reports demonstrate substantial translational interest in phage-based therapies for difficult-to-treat and antibiotic-resistant infections [39,40]. In this context, the study by Pirnay and colleagues provides real-world evidence from 100 consecutive cases of personalized bacteriophage therapy in difficult-to-treat infections, supporting clinical feasibility but not directly evaluating persistence-specific endpoints. By contrast, ongoing randomized studies registered on ClinicalTrials.gov, such as NCT07619924 [40], may provide stronger prospective clinical evidence, although they are likewise not specifically designed to assess persister biology, PPE, or PW endpoints. Overall, they support the feasibility of integrating non-conventional anti-infective strategies into antimicrobial pharmacology.

**Figure 5:**
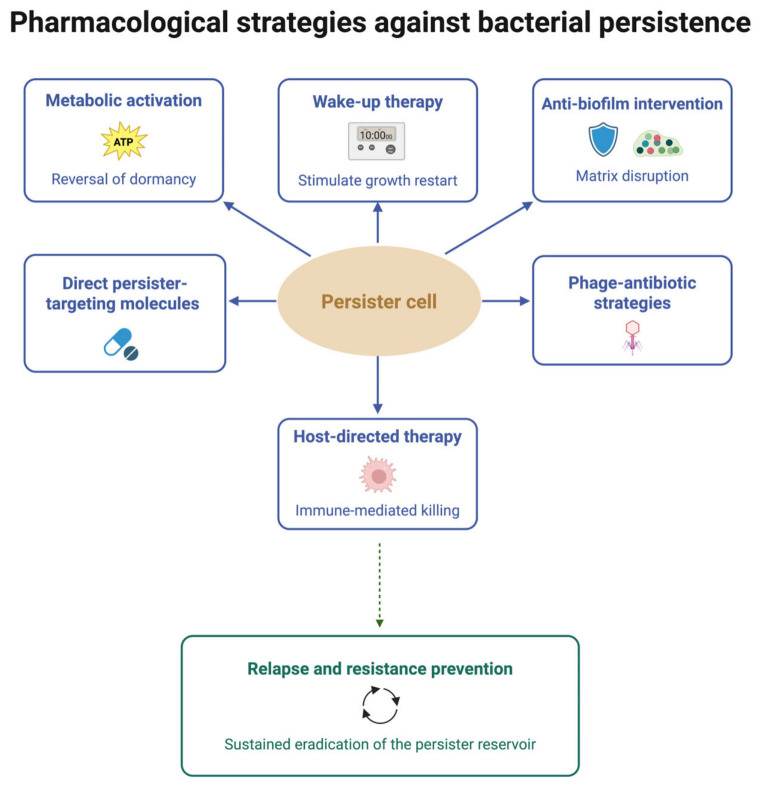
Pharmacological strategies against bacterial persistence. Five therapeutic strategies (metabolic activation, wake-up therapy, anti-biofilm intervention, direct persister-targeting molecules, and phage-antibiotic strategies) converge on the persister cell to overcome phenotypic antibiotic tolerance. Host-directed therapy complements these approaches through immune-mediated killing. Together, they aim to achieve sustained eradication of the persister reservoir to prevent relapse and the selection of resistance. ATP, adenosine triphosphate. Figure created with BioRender (https://www.biorender.com, accessed on 22 June 2026).

Viewed through the lens of persistence, phage therapy should not be considered merely an alternative to antibiotics but rather a complementary tool capable of addressing biological and pharmacological barriers that limit antibiotic efficacy. Future precision antimicrobial strategies may increasingly combine optimized antibiotic exposure, PPE, anti-biofilm interventions, host-directed therapies, and phage-based approaches to achieve durable bacterial eradication and reduce persistence-driven relapse (Figure 5).

## 11. AI and Precision Anti-Persistence Therapy

The management of bacterial persistence represents a uniquely complex pharmacological challenge. Unlike conventional antimicrobial resistance, which is often characterized by relatively discrete microbiological endpoints such as susceptibility profiles and resistance determinants, persistence emerges from the dynamic interaction of multiple biological, pharmacokinetic, pharmacodynamic, and host-related factors. This complexity creates a compelling rationale for the application of AI and machine-learning approaches to antimicrobial decision-making.

In recent years, AI has demonstrated significant potential across several areas of antimicrobial research. One of the most notable examples was provided by Stokes and colleagues, who trained a deep neural network to predict antibacterial activity across millions of compounds and identified halicin, a molecule structurally divergent from conventional antibiotics, with bactericidal activity against several clinically relevant pathogens, including carbapenem-resistant *Enterobacteriaceae* and *Mycobacterium tuberculosis* [41]. This work established the proof of concept that AI can accelerate antibiotic discovery by identifying structurally novel candidates overlooked by conventional screening approaches. At the same time, Theuretzbacher and colleagues highlighted the ongoing limitations of the antibacterial development pipeline and stressed the importance of innovative methodologies to expand therapeutic options for difficult-to-treat infections [133].

Subsequent studies extended this foundational work along three complementary directions. Wong and colleagues applied explainable graph neural networks to screen 39,312 compounds and predict antibacterial activity for over 12 million candidates, identifying a structural class of antibiotics with selective activity against methicillin-resistant Staphylococcus aureus and vancomycin-resistant enterococci; crucially, the use of substructure-based chemical rationales rendered model predictions interpretable, addressing a central translational limitation of black-box approaches [42]. Scalia and colleagues at Genentech developed GNEprop, a deep learning model trained on approximately two million phenotypic screening datapoints against a sensitized Escherichia coli strain, which enabled virtual screening of over 1.4 billion synthetically accessible compounds and achieved a 90-fold improvement in hit rate over conventional high-throughput screening; many identified candidates were structurally dissimilar to known antibiotics and had validated biological targets [134]. Olayo-Alarcon, Müller and colleagues introduced MolE, a self-supervised framework that learns task-independent molecular representations from unlabeled chemical structures, requiring minimal compound-specific training data; applied to antimicrobial discovery, MolE identified three human-targeted drugs as de novo growth inhibitors of Staphylococcus aureus, illustrating the potential of low-data AI strategies to prioritize previously overlooked antimicrobial scaffolds from existing chemical libraries, a particularly attractive approach given the current scarcity of validated compound–persister activity datasets [135]. Taken together, these four studies delineate a rapidly maturing AI toolkit for antibacterial discovery whose explainability, scalability, and low-data adaptability might be highly relevant to, but have not yet been specifically validated for, the identification of growth-independent bactericidal agents capable of targeting dormant persister populations.

Complementing these compound-discovery approaches, Benedetto and colleagues demonstrated that machine learning applied to routinely collected clinical and microbiological data from two Italian hospital centers, encompassing 15,581 bacterial isolates from 9966 patients, can accurately predict antibiotic susceptibility profiles before culture results are available [43]. XGBoost models achieved area-under-the-receiver-operating-characteristic-curve values of up to 0.946 for Pseudomonas aeruginosa, 0.941 for Klebsiella pneumoniae, and 0.891 for Staphylococcus aureus, pathogens strongly associated with biofilm formation and clinical persistence, with a potential reduction in treatment delays of up to 48 h compared with conventional diagnostic workflows. From a persistence-pharmacology perspective, these results could theoretically translate directly into earlier attainment of effective antimicrobial exposure, reducing the time spent within the PW and aligning clinical practice with the first pillar of the Six-Pillar Pharmacological Persistence Prevention Model proposed in this review. However, this interpretation remains inferential, because the model was designed to predict antimicrobial susceptibility rather than persistence-specific outcomes.

While AI-driven antibiotic discovery represents an important advance, the potential role of AI in persistence-oriented therapy may extend far beyond identification of new antimicrobial agents. Machine-learning approaches have also been applied to predict bacterial tolerance and persistence phenotypes from genomic and transcriptomic data and to model time-kill dynamics and persister-fraction kinetics in silico, suggesting a specific translational role for computational methods in persistence-oriented pharmacology [54].

The survival of persister populations depends not only on pathogen characteristics but also on antibiotic exposure, tissue pharmacology, infection-site conditions, bacterial physiological state, host immune responses, and treatment history. These interacting variables generate highly complex datasets that frequently exceed the capacity of traditional clinical decision-making frameworks. From a precision-medicine perspective, AI may serve as an integrative platform capable of combining diverse sources of information into clinically actionable predictions. Relevant variables could include pathogen species, antimicrobial susceptibility profiles, resistance determinants, whole-genome sequencing data, time–kill kinetics, persister fractions, biofilm-forming capacity, infection localization, tissue penetration characteristics, organ function, prior antimicrobial exposure, immune status, inflammatory biomarkers, microbiome composition, therapeutic drug monitoring (TDM) data, and previous episodes of treatment failure or relapse.

Importantly, persistence itself may ultimately be conceptualized as a predictive phenotype rather than a purely microbiological phenomenon. Machine-learning models trained on large clinical and experimental datasets may be capable of identifying patterns associated with increased risk of persistence-driven treatment failure, even when conventional susceptibility testing predicts favorable outcomes. Such models could facilitate early identification of patients at high risk of relapse and support more individualized therapeutic strategies.

Current model-informed precision dosing approaches already incorporate Bayesian forecasting and population PK models to optimize antibiotic exposure [47]. Future AI-enabled systems could extend this framework by simultaneously incorporating target-site pharmacology, biofilm characteristics, host inflammatory status, and persistence-related biomarkers. Such tools may facilitate estimation of the probability of achieving not only conventional PK/PD targets but also the proposed PPE, provided that PPE is experimentally operationalized and validated as a measurable pharmacodynamic endpoint.

Similarly, AI may contribute to optimization of therapeutic combinations and treatment sequencing. Because persistence frequently involves heterogeneous bacterial populations occupying distinct physiological states, selecting the optimal combination of antibiotics, metabolic activators, anti-biofilm agents, phages, or host-directed therapies represents a highly complex decision problem. Computational approaches may help identify treatment strategies most likely to achieve durable bacterial eradication while minimizing toxicity and the selection of resistance.

Another potentially transformative application is the development of predictive models for relapse risk. Current clinical decision-making often relies on static microbiological endpoints obtained at a single time point. In contrast, persistence is inherently dynamic and may evolve throughout the course of infection. AI systems capable of integrating longitudinal clinical data, biomarker trajectories, microbiological findings, and treatment-response patterns could provide individualized estimates of relapse probability and support adaptive therapeutic interventions.

AI may also improve the design and interpretation of clinical trials targeting persistence. One of the major challenges in anti-persister drug development is the difficulty of identifying patients most likely to benefit from novel interventions. AI-driven patient stratification could enrich clinical trials by identifying subjects at high risk of persistence-associated treatment failure, thereby increasing statistical power and improving the evaluation of emerging anti-persister therapies.

Nevertheless, enthusiasm regarding AI should be balanced by recognition of important limitations. Reliable predictive models require large, high-quality, and clinically representative datasets, which remain scarce in the field of persistence. Furthermore, clinical practice does not routinely measure many persistence-related variables, such as dormancy depth, persister fraction, target-site exposure, and biofilm burden. The absence of standardized persistence biomarkers currently represents a major obstacle to model development and validation. Importantly, none of the AI frameworks reviewed herein, whether applied to compound discovery [41,42,134,135], clinical resistance prediction [43], or antimicrobial pharmacokinetics, has been prospectively validated against persistence-specific endpoints such as post-treatment regrowth, persister fraction reduction, or relapse prevention. Bridging this gap will require dedicated study designs that incorporate persistence-related outcomes as primary endpoints.

Consequently, the most realistic near-term role of AI is unlikely to be autonomous treatment selection. Instead, AI should be viewed as a clinical decision-support tool capable of assisting clinicians in risk stratification, PK/PD simulation, treatment individualization, and identification of patients at increased risk of persistence-driven relapse. In this role, AI complements rather than replaces clinical expertise.

Precision anti-persistence therapy will likely depend on the convergence of antimicrobial pharmacology, systems biology, TDM, target-site pharmacology, and AI. By integrating these traditionally separate domains, AI may provide the computational framework necessary to translate the growing complexity of persister biology into actionable clinical strategies. Such an approach would represent a natural evolution from conventional antimicrobial stewardship toward a new paradigm of precision antimicrobial stewardship, focused not only on preventing resistance but also on preventing persistence-driven treatment failure and relapse.

## 12. Six-Pillar Pharmacological Persistence Prevention Model

**Figure 6:**
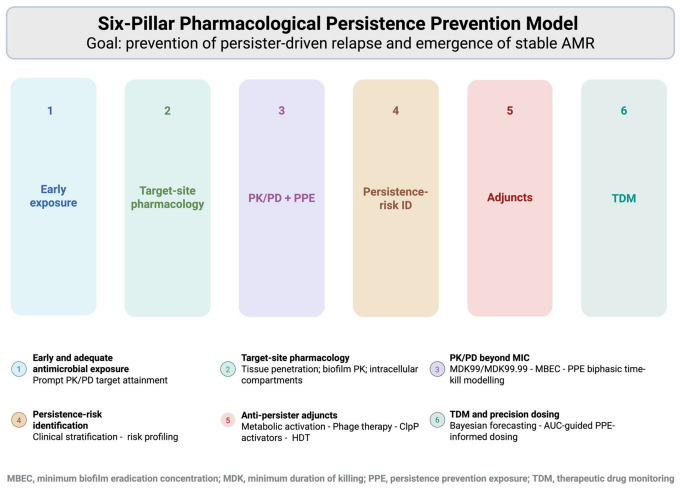
The Six-Pillar Pharmacological Persistence Prevention Model. Schematic representation of six synergistic intervention domains for preventing persister-driven relapse and stable AMR emergence: early antimicrobial exposure (1); target-site pharmacology (2); PK/PD optimization beyond MIC, including PPE (3); persistence-risk stratification (4); anti-persister adjuncts (5); and TDM with model-informed precision dosing (6). AMR, antimicrobial resistance; ClpP, caseinolytic protease P; HDT, host-directed therapy; MBEC, minimum biofilm eradication concentration; MDK, minimum duration for killing; PK, pharmacokinetic; PK/PD, pharmacokinetic/pharmacodynamic; PPE, persistence prevention exposure; TDM, therapeutic drug monitoring. Figure created with BioRender (https://www.biorender.com, accessed on 22 June 2026).

The Six-Pillar Pharmacological Persistence Prevention Model integrates the conceptual and clinical dimensions developed throughout this review into a coherent, implementable framework for anti-persistence-oriented antimicrobial therapy. The model is organized around six distinct but interrelated domains, each addressing a critical determinant of persister survival and therapeutic failure. Together, they represent a shift from susceptibility-centered treatment toward a strategy focused on durable bacterial eradication [6,14] (Figure 6).

Pillar 1. Early and Adequate Antimicrobial Exposure establishes that prompt attainment of therapeutic concentrations is a prerequisite for preventing the pharmacological conditions that permit persister selection. Delayed or subtherapeutic exposure during the initial phase of therapy creates a window of vulnerability in which bacterial populations can adapt and enter dormant states, increasing the likelihood of subsequent treatment failure [44,50].

Pillar 2. Target-Site Pharmacology recognizes that plasma PK/PD target attainment is a necessary but insufficient condition for eradicating persister reservoirs. Effective therapy requires adequate drug exposure at the actual site of infection, including within biofilms, intracellular compartments, avascular tissue, and other pharmacological sanctuaries [12,31,50]. Source-control interventions are an integral component of this pillar (Section 7).

Pillar 3. PK/PD Optimization Beyond MIC extends classical pharmacodynamic indices to persistence-relevant parameters, as operationalized through the PPE concept. Integration of MDK_99_/MDK_99.99_, persister fraction analysis, MBEC, and biphasic time-kill modeling provides a multidimensional exposure target that goes beyond growth inhibition [6,18,21] (Section 5; Table 3).

Pillar 4. Persistence-Risk Identification applies the clinical framework outlined in Table 5 to systematically identify infection settings in which bacterial persistence is likely to be pharmacologically relevant. Prosthetic joint infections, osteomyelitis, endocarditis, chronic respiratory infections, recurrent urinary tract infections, tuberculosis, and device-associated infections all share features (biofilm, impaired penetration, intracellular niches, or high bacterial burden) that increase the probability of persistence-driven treatment failure [7,14,71].

Pillar 5. Anti-Persister Adjuncts encompasses the emerging therapeutic strategies reviewed in Section 10, including metabolic activation, wake-up therapy, direct persister-targeting molecules, host-directed therapy, biomaterial-based delivery systems, and phage-antibiotic combinations. These interventions are not intended to replace conventional antimicrobial therapy but to complement it by specifically targeting bacterial subpopulations that evade standard antibiotic killing [22,35,36,50] (Table 4).

Pillar 6. TDM and Precision Dosing translates the theoretical constructs of PPE and the PW into clinical practice through individualized TDM. By incorporating Bayesian forecasting, model-informed precision dosing, and target-site pharmacokinetic estimates, this pillar provides a practical mechanism for minimizing exposure within persistence-promoting concentration ranges while optimizing durable bacterial eradication [31,33,44,47] (Section 8). The integration of AI into this pillar, as discussed in Section 11, may further enhance the capacity to predict persistence risk and individualize therapy once persistence-specific endpoints and biomarkers are standardized and prospectively validated [41,42,43].

Taken together, the Six-Pillar Model represents a translational bridge between experimental persister biology and antimicrobial stewardship, providing a structured framework for applying persistence-oriented pharmacology to clinical decision-making. Although prospective validation is required before the model can be considered an established clinical algorithm, it offers a practical conceptual architecture for integrating PK/PD optimization, target-site pharmacology, TDM, anti-persister adjuncts, source control, and AI-enabled precision stewardship into future strategies to prevent persistence-driven treatment failure and relapse.

## 13. Limitations and Challenges for Clinical Translation

Despite the growing recognition of bacterial persistence as a major contributor to treatment failure, relapse, and chronic infection, several important limitations currently hinder the translation of persister biology into routine clinical practice [7,14]. These limitations should be carefully considered when interpreting both the existing evidence and the conceptual frameworks proposed in this review.

First, much of the current understanding of persistence remains derived from in vitro experiments, animal models, and highly controlled laboratory systems [6,34]. Although these studies have substantially advanced knowledge of persister biology, their relevance to complex human infections is not always straightforward. The physiological conditions encountered during clinical infection, including host immune responses, tissue heterogeneity, biofilm formation, altered pharmacokinetics, and polymicrobial interactions, are difficult to reproduce experimentally [31,50]. Consequently, the extent to which findings from preclinical models accurately predict clinical outcomes remains uncertain.

Second, the field continues to lack standardized and widely accepted methods for measuring persistence. Unlike antimicrobial resistance, which can be quantified using established susceptibility testing procedures, persistence remains operationally defined through a variety of experimental approaches that differ substantially across studies [6,13]. Time-kill assays, persister fraction measurements, MDK_99_ and MDK_99.99_ determinations, biofilm eradication models, and regrowth experiments all provide valuable information, yet no single method has emerged as a universally accepted clinical standard [6,18,21]. This methodological heterogeneity complicates comparisons among studies and limits the development of reproducible persistence-related endpoints.

Third, routine clinical microbiology remains largely focused on resistance detection rather than persistence characterization. Minimum inhibitory concentration (MIC) testing, while indispensable for guiding antimicrobial therapy, provides little information regarding bacterial dormancy, killing kinetics, biofilm-associated survival, intracellular persistence, or the probability of post-treatment regrowth [6,13,14]. As a result, patients may experience persistence-driven relapse despite apparently favorable susceptibility profiles and achievement of conventional PK/PD targets.

A related limitation is the lack of validated biomarkers to identify patients at increased risk of persistence-associated treatment failure. Unlike resistance, which can often be linked to specific genetic determinants, persistence reflects a dynamic physiological state influenced by bacterial, host, pharmacological, and environmental factors [15,50]. The lack of clinically accessible biomarkers currently limits risk stratification, patient selection, and therapeutic personalization.

Persister formation and survival are influenced by pathogen species, bacterial genotype, infection site, host immune status, antibiotic class, dosing regimen, tissue pharmacology, and local microenvironmental conditions [7,34,50]. Consequently, persistence should not be regarded as a uniform biological phenomenon. Strategies effective against one pathogen or infection type may prove ineffective in others, making universal anti-persister approaches unlikely.

The conceptual frameworks proposed in this review, including the PW and PPE, should also be interpreted with appropriate caution. These constructs are intended as pharmacological models designed to facilitate integration of persister biology into PK/PD thinking. At present, however, they remain theoretical frameworks rather than validated clinical parameters. Prospective experimental studies and clinical investigations will be required to determine whether such concepts can be translated into measurable, reproducible, and clinically actionable endpoints.

An additional misconception that warrants attention is the assumption that persistence can be overcome simply by increasing antibiotic dose or extending treatment duration. Persistence frequently reflects altered bacterial physiology, reduced target engagement, biofilm protection, intracellular localization, or inadequate target-site exposure rather than insufficient systemic drug concentration alone [8,14,31]. Consequently, indiscriminate escalation of antibiotic exposure may increase toxicity, disrupt host microbiota, and amplify ecological selection pressure without necessarily improving eradication of persister populations [44,129]. Effective anti-persister therapy will therefore require mechanistically informed optimization rather than empirical intensification of treatment.

Several emerging therapeutic approaches discussed in this review, including metabolic activation, wake-up therapy, direct persister-targeting molecules, host-directed therapies, nanomedicine, and phage-based interventions, also face important translational challenges. Most of these approaches are still in the preclinical or early clinical stages of development, and robust evidence demonstrating improved patient outcomes remains limited [34,36,50]. Furthermore, many published studies evaluate microbiological endpoints without specifically assessing relapse prevention, durable eradication, or reduction in persister populations. This issue is particularly evident in the field of bacteriophage therapy. Although an increasing number of clinical trials and compassionate-use experiences support the feasibility of phage-based interventions, persistence-specific outcomes are rarely incorporated into study designs [39,40]. Particularly, the study by Pirnay and colleagues provides real-world evidence from 100 consecutive cases of personalized bacteriophage therapy in difficult-to-treat infections, whereas NCT07619924 represents an ongoing randomized study of a phage cocktail for multidrug-resistant bacterial skin infections; however, neither is specifically designed around persister fraction reduction, PPE, or PW endpoints [39,40]. Similar limitations apply to many emerging anti-persister strategies, where relapse prevention is often inferred rather than directly measured.

Many of the variables most relevant to persistence, including biofilm burden, dormancy depth, persister fractions, and local antibiotic exposure, are not routinely collected in clinical practice [31,54]. As a result, development of predictive models capable of supporting precision anti-persistence therapy remains an important future objective rather than an immediately achievable reality [43,47].

Taken together, these limitations highlight that bacterial persistence remains an evolving field situated at the interface of experimental microbiology and clinical pharmacology. While significant progress has been made in understanding the biological basis of persistence and in developing innovative therapeutic concepts, substantial work remains before these advances can be fully translated into routine patient care. Recognition of these challenges is essential to ensure that future anti-persister strategies are developed within a rigorous and evidence-based framework.

## 14. Conclusions and Future Perspectives

Bacterial persistence represents one of the most important conceptual challenges to the traditional paradigm of antimicrobial therapy. Unlike resistant bacteria, persister cells remain genetically susceptible to antibiotics yet survive exposure through reversible physiological adaptations that transiently reduce antibiotic-mediated killing [6,27]. Although persistence does not constitute heritable resistance, its clinical consequences can be profound, contributing to treatment failure, chronic infection, relapse, prolonged antibiotic exposure, and ultimately creating opportunities for the emergence and selection of genetically resistant populations [27,28,29].

The growing recognition of persistence highlights a fundamental limitation of conventional antimicrobial pharmacology. Current therapeutic strategies are largely built around susceptibility testing and achievement of PK/PD targets derived from the MIC. While these approaches remain indispensable, they primarily address the inhibition and killing of actively growing bacteria and provide limited insight into the survival of dormant, biofilm-associated, intracellular, or otherwise protected bacterial subpopulations [6,13,14].

Throughout this review, we have argued that bacterial persistence should be viewed not only as a microbiological phenomenon but also as a pharmacological challenge arising from the interaction among bacterial physiology, antimicrobial exposure, infection-site conditions, and host biology [15,31,50]. This perspective places persistence at the intersection of pharmacokinetics, pharmacodynamics, tissue pharmacology, biofilm biology, immunology, and systems medicine.

To facilitate integration of persister biology into antimicrobial pharmacology, we proposed the concepts of the PW and PPE. These frameworks are not intended to replace established PK/PD principles or susceptibility testing. Importantly, PPE should not be interpreted as a validated breakpoint or a new susceptibility metric. At present, it remains a hypothesis-generating and testable pharmacological construct that requires experimental validation.

Future studies should evaluate PPE alongside complementary persistence-related metrics, including MDK_99_ and MDK_99.99_ measurements, quantification of the persister fraction, biphasic killing dynamics, biofilm eradication parameters, relapse models, and target-site pharmacokinetic assessments [6,13,18]. Such approaches may help determine whether persistence-oriented exposure targets can improve prediction of long-term therapeutic outcomes.

Metabolic activation, wake-up therapies, direct persister-targeting molecules, anti-biofilm approaches, advanced biomaterials, host-directed therapies, and phage–antibiotic combinations collectively demonstrate that future infection management may increasingly focus on eliminating bacterial reservoirs that survive conventional treatment rather than simply increasing antibiotic exposure [22,35,36,50]. These approaches emphasize the importance of targeting bacterial physiological state, infection-site ecology, and host–pathogen interactions alongside traditional antimicrobial susceptibility.

Future progress in the field will depend on several key developments. First, standardized experimental and clinical methods for measuring persistence must be established [6,21]. Second, clinically relevant biomarkers that can identify persistence-prone infections and predict relapse risk are urgently needed. Third, target-site pharmacology should be more systematically incorporated into antimicrobial development and therapeutic monitoring [31]. Fourth, prospective clinical trials should move beyond conventional microbiological endpoints and evaluate outcomes directly related to persistence, including post-treatment regrowth, recurrence, and durable eradication.

By integrating microbiological, pharmacokinetic, pharmacodynamic, immunological, and clinical variables, computational tools may eventually enable individualized prediction of the risk of persistence and support precision anti-persistence therapy [43,47,54]. Such approaches could facilitate patient stratification, optimize treatment selection, improve TDM, and accelerate development of novel anti-persister interventions. However, their clinical utility will depend on the availability of standardized persistence-related endpoints, high-quality longitudinal datasets, and prospective validation against relapse or durable-eradication outcomes.

For decades, the primary objective of antimicrobial therapy has been to suppress bacterial growth and prevent the emergence of resistance. The next frontier may be preventing persistence-driven relapse. Achieving this goal will require a shift from a purely susceptibility-centered perspective toward a more integrated framework that incorporates bacterial physiology, target-site exposure, host biology, and long-term eradication outcomes. In this evolving landscape, the future of anti-infective therapy may be defined not only by the ability to kill bacteria during treatment but also by the ability to prevent their return after treatment has ended. Moving from resistance-oriented therapy to persistence-oriented pharmacology may therefore represent one of the most important challenges and opportunities in the next generation of antimicrobial research and clinical practice.

## Figures and Tables

**Table 2:** Pharmacological distinction between resistance, tolerance, persistence, and VBNC states.

Feature	Resistance	Tolerance	Persistence	VBNC State
MIC	Increased	Usually, unchanged	Usually, unchanged	May be difficult to measure
Population affected	Whole resistant population	Most/all cells	Small subpopulation	Variable
Genetic basis	Usually, genetic	Often non-genetic	Non-genetic phenotypic state	Stress-induced physiological state
Regrowth after stress removal	Resistant growth persists	Growth resumes	Growth resumes	Often no growth under standard culture
Core assay	MIC testing	MDK/time-kill	Biphasic time-kill/persister fraction	Viability assays beyond culture
Pharmacological implication	Alternative active drug	Optimize duration and exposure	Anti-persister strategy	Diagnostic and eradication challenge

**Table 3:** Operational relationship between PPE and existing tolerance/persistence metrics.

Metric	What It Measures	Limitation	How PPE Relates to It
MIC	Growth inhibition under standardized conditions	Does not measure killing, dormancy, or regrowth	PPE starts from MIC but extends beyond it
MDK_99_/MDK_99.99_	Time required to kill 99% or 99.99% of cells	Population-level killing metric; may not include target-site PK	PPE uses MDK as a killing-time input
Persister fraction	Residual surviving subpopulation after exposure	Assay-dependent and model-dependent	PPE aims to reduce or prevent this fraction
Biphasic time-kill modeling	Separation of rapid killing and survival tail	Requires standardized dynamic assays	PPE targets the elimination of the survival tail
MBEC	Biofilm eradication concentration	The biofilm method varies by model	PPE incorporates MBEC when biofilm is the relevant niche
Target-site PK	Drug exposure at infection site	Difficult to measure clinically	PPE is infection-site rather than plasma-only exposure

**Table 4:** Mechanism-driven combination strategies against persistence.

Approach	Rationale	Potential Context	Main Limitation
Beta-lactam + aminoglycoside	Cell-wall perturbation plus concentration-dependent killing	Selected severe infections	Nephrotoxicity and ototoxicity
Daptomycin + beta-lactam	Membrane targeting plus cell-wall modulation	Selected Gram-positive infections	Limited persistence-specific trials
Rifampicin combinations	Biofilm and intracellular activity	Selected staphylococcal device infections	Resistance if used alone
Antibiotic + metabolic adjuvant	Reactivates metabolism and antibiotic uptake	Experimental persister eradication	Preclinical and pathogen-specific
Antibiotic + anti-biofilm agent	Disrupts protective niche	Device/biofilm infections	Model heterogeneity
Phage + antibiotic	Distinct killing mechanisms and possible biofilm effects	Difficult-to-treat or MDR infections	Personalization and regulation

**Table 5:** Clinical settings where bacterial persistence may be pharmacologically relevant.

Clinical Setting	Persistence-Promoting Factor	Pharmacological Implication
Prosthetic joint infection	Foreign material and biofilm	Source control, biofilm-active combinations when appropriate
Endocarditis	High bacterial burden and vegetations	Bactericidal exposure and prolonged therapy
Osteomyelitis	Poor penetration and necrotic bone	Target-site exposure and source control
Chronic lung infection	Mucus and biofilm barriers	Local exposure and anti-biofilm approaches
Recurrent urinary tract infection	Reservoirs and intracellular niches	Relapse prevention and tissue exposure
Tuberculosis/intracellular infection	Dormancy and macrophage localization	Multidrug therapy and host-directed strategies
Catheter-associated infection	Device biofilm	Device management/source control
Abscess	Low pH and poor penetration	Drainage plus optimized antibiotic exposure

## Data Availability

No new data were created or analyzed in this study. Data sharing is not applicable.

## Abbreviations

ADEP	Acyldepsipeptide
AI	Artificial intelligence
AMR	Antimicrobial resistance
ATP	Adenosine triphosphate
AUC	Area under the concentration-time curve
CDC	Centers for Disease Control and Prevention
Cmax	Maximum concentration
EPS	Extracellular polymeric substance
fT > MIC	Fraction of time that free drug concentration remains above the minimum inhibitory concentration
GLASS	Global Antimicrobial Resistance and Use Surveillance System
MBEC	Minimum biofilm eradication concentration
MDK_99_	Minimum duration for killing 99% of a bacterial population
MDK_99.99_	Minimum duration for killing 99.99% of a bacterial population
MIC	Minimum inhibitory concentration
MPC	Mutant prevention concentration
MSW	Mutant selection window
PD	Pharmacodynamics
PK	Pharmacokinetics
PK/PD	Pharmacokinetics/pharmacodynamics
PPE	Persistence Prevention Exposure
PRISMA	Preferred Reporting Items for Systematic Reviews and Meta-Analyses
SANRA	Scale for the Assessment of Narrative Review Articles
TDM	Therapeutic drug monitoring
VBNC	Viable but non-culturable
WHO	World Health Organization
